# *Salvia desoleana* Atzei et Picci Steam-Distillation Water By-Products as a Source of Bioactive Compounds with Antioxidant Activities

**DOI:** 10.3390/foods14132365

**Published:** 2025-07-03

**Authors:** Valentina Masala, Gabriele Serreli, Antonio Laus, Monica Deiana, Adam Kowalczyk, Carlo Ignazio Giovanni Tuberoso

**Affiliations:** 1 Department of Life and Environmental Sciences, University of Cagliari, Cittadella Universitaria di Monserrato, S.P. Monserrato-Sestu km 0.700, 09042 Monserrato, Italy; valentina.masala2@unica.it; 2 Department of Biomedical Sciences, University of Cagliari, Cittadella Universitaria di Monserrato, S.P. Monserrato-Sestu km 0.700, 09042 Monserrato, Italy; gabriele.serreli@unica.it (G.S.); mdeiana@unica.it (M.D.); 3 Department of Life Sciences, University of Modena and Reggio Emilia, 41125 Modena, Italy; antonio.laus@unimore.it; 4 Department of Pharmacognosy and Herbal Medicines, Faculty of Pharmacy, Wroclaw Medical University, 50-556 Wrocław, Poland; adam.kowalczyk@umw.edu.pl

**Keywords:** LC-MS/MS, HPLC-PDA, metabolic profiling, molecular docking, iNOS, Keap1-Nrf2, NOX, glutathione, cysteine-cystine, reactive oxygen species

## Abstract

In this study, water residue obtained from *Salvia desoleana* Atzei et Picci steam distillation was evaluated for its antioxidant activity in vitro using different experimental models. In particular, the study evaluated the antiradical and antioxidant activity of *Salvia desoleana* extracts using CUPRAC, FRAP, DPPH^•^, and ABTS^•+^ assays; and tested ROS scavenging activity in Caco-2 cell cultures. Phenolic compounds were identified by (HR) LC-ESI-QTOF MS/MS and quantified with HPLC-PDA. Furthermore, Keap1-Nrf2, iNOS, and NOX enzymes involved in oxidative stress and antioxidant defences were the targets of molecular docking on key polyphenols. Hydroxycinnamic acids and flavonoids are the most important classes of compounds detected in the extracts. Among these compounds, the most significant was rosmarinic acid, followed by caffeic acid, luteolin glucuronide, and methyl rosmarinate. Although all extracts have shown encouraging results, the ethanolic extract solubilised with water (SEtOHA) was the one with the highest hydroxycinnamic acid content and total phenol content (518.64 ± 5.82 mg/g dw and 106.02 ± 6.02 mg GAE/g dw), as well as the highest antioxidant and antiradical activity. The extracts have shown anti-inflammatory activity by inhibiting NO release in LPS-stimulated Caco-2 cells. Finally, the in silico evaluation against the three selected enzymes showed interesting results for both numerical affinity ranking and predicted ligand binding models. The outcome of this study suggests this by-product as a possible ally in counteracting oxidative stress, as established by its favourable antioxidant compound profile, thus suggesting an interesting future application as a nutraceutical.

## 1. Introduction

Polyphenol intake is associated with beneficial effects on ageing and a lower incidence of age-related diseases in populations of the Blue Zones [1]. Sardinia (Italy) is one of the most well-known Blue Zones in the world due to the longevity of its population and is characterised by a high percentage of male centenarians [2]. These peculiarities are closely related to genetic traits, lifestyle, quality, and quantity of food intake. Vegetables represent an important traditional food source in the Sardinian diet, consisting of many species from fava beans to fennel, from thistles to *Salvia* spp. [3]. The genus *Salvia*, which belongs to the *Lamiaceae* family, comprises more than 1000 species and is distributed all around the globe [4]. Medicinal and aromatic plants are increasingly utilised in the food industry due to their bioactive compounds, particularly essential oils, which exhibit antioxidant, antimicrobial, and preservative properties. To increase shelf life, prevent microbiological development, and enhance sensory qualities, these natural additions are used in a variety of food products, including meat, dairy, baked goods, and beverages. Their application provides an alternative to artificial preservatives and is in line with the rising desire for functional and clean-label goods [5]. Besides the traditional usage as aromatic plants for essential oil production and the importance in the food industry as a spice for giving a particular aroma and taste, the interest in these species has constantly increased due to their nutraceutical and pharmacological properties [5]. *Salvia desoleana* Atzei et Picci, an indigenous species from the island of Sardinia, has aroused ever-increasing interest over the years. It is an evergreen perennial herbaceous plant with many rhizomatous caules that thrives in sunny regions with calcareous, granitic, and granitic-porphyritic soil types. *S. desoleana* blooms between mid-May and mid-July, and between August and November, more efflorescence may occasionally occur, although it is restricted to fresh axillary buds. It is of notable interest due to its intense and persistent aroma, which is already present during the vegetative stage, as well as for the high biomass yield of both distillable material and herbal drug it can provide [6,7,8]. For many years, this sage species was thought to be a variant of *S. sclarea* due to the similar morphological, systematic, and phytochemical relationships. However, further studies have attributed this plant to a new, different species [6]. Since ancient times, *S. desoleana* has been widely utilised in traditional medicine. An infusion having antipyretic, antispasmodic, hypertensive, stimulant, tonic, and astringent qualities was made from its stems and leaves and is still used today [9]. The essential oil content varies between 0.5 and 2.5% depending on the plant part used, harvest season, and cultivation soil [8]. It contains linalyl acetate, germacrene D, α-terpinyl acetate, 1,8-cineole, linalool, and β-pinene, which are responsible for some of its medicinal properties, such as antimicrobial, antifungal, antiviral, and anti-inflammatory ones [8,9,10,11,12]. So far, the main commercial application of *S. desoleana* is the production of the valuable essential oil by steam- and hydro-distillation procedures [10]. Steam distillation is a typical separation process, particularly useful for isolating temperature-sensitive compounds, including essential oils, which can decompose at standard distillation temperatures [13]. The technique has been widely used to extract essential oils from aromatic plants, including *Eucalyptus globulus* and *Mentha arvensis* [14]. However, it has several limitations, such as potentially lower yields than modern procedures and the loss of volatile components throughout the processing. The process is time-consuming, and some constituents may degrade due to high temperatures. Notwithstanding these shortcomings, steam distillation is still popular because it is inexpensive and produces high-quality essential oils [13,15]. Steam distillation produces significant by-products, such as hydrosols (aromatic fluids) and discarded plant biomass, in addition to essential oils, both of which present worthwhile prospects for environmental and industrial use. Applications in cosmetics, aromatherapy, and food preservation are possible due to the water-soluble aromatic compounds found in hydrosols, the aqueous condensate co-produced during distillation, which also exhibit antimicrobial, anti-inflammatory, and antioxidant properties [16]. This activity produces a large amount of by-products, represented by hydrolates, water residues, and solid residues. A study on these by-products from the hydro-distillation of some *Lamiaceae* plants (sage, rosemary, and bay laurel) [17] reported a noteworthy presence of bioactive substances, including polyphenols and oxygenated compounds, which have important uses in medicines, cosmetics, and nutraceuticals. The by-products of hydro-distillation contain a multitude of bioactive chemicals that can be used for a variety of purposes, including antioxidant, antibacterial, and enzyme inhibition. For instance, aromatic plant by-products such as thyme, oregano, and basil are high in antioxidants and enzyme inhibitors. These chemicals have strong radical-scavenging abilities and can inhibit enzymes, including acetylcholinesterase, tyrosinase, amylase, and glucosidase [18]. Moreover, hydrosols include slight levels of essential oils and have valuable antibacterial capabilities [19]. These cases are representative of a larger trend in the food and agriculture sectors towards the valorisation of by-products to improve sustainability, lessen the impact on the environment, and produce goods with added value [20].

Taking into account these observations and the fact that *S. desoleana* has not yet been investigated for its phenolic composition, and to promote a more sustainable and resource-efficient strategy, the present research aims to investigate the aqueous fraction residual from its steam distillation. Qualitative and quantitative analyses of bioactive compounds were performed using (HR) LC-ESI-QTOF MS/MS in the negative and positive ion modes and HPLC-PDA analysis. This by-product, typically discarded, was found to contain a rich profile of phenolic compounds, especially belonging to the hydroxycinnamic acid and flavonoid classes. These compounds were further evaluated for their antioxidant and radical-scavenging activities, revealing significant bioactive potential. The total polyphenol (TP) content was determined via Folin–Ciocalteu’s assay, and antioxidant activity (AA) was assessed by the DPPH^•^ (2,2-diphenyl-1-picrylhydrazyl radical), ABTS^•+^ (2,2-azinobis-(3-ethylbenzothiazoline-6-sulphonic acid)), FRAP (ferric reducing antioxidant power), and CUPRAC (cupric-ion-reducing antioxidant capacity) assays. Furthermore, the ability to scavenge reactive oxygen species (ROS) was also tested in intestinal differentiated Caco-2 cell cultures. In addition, the molecular docking of target polyphenols was performed within the active sites of Kelch-like ECH-associated protein 1 (Keap1), inducible nitric-oxide synthase (iNOS), and NADPH oxidase 2 (NOX2) enzymes that are responsible for the improvement of cellular antioxidant defences, the release of nitric oxide (NO), and the production of ROS. The novelty of this work lies in the valorisation of a previously unexplored by-product, demonstrating that the aqueous phase from steam distillation can serve as a sustainable and valuable source of natural antioxidants. This creates new opportunities for the reuse of aromatic plant by-products in high-value uses, such as functional components in the cosmetic, pharmaceutical, and nutraceutical sectors.

## 2. Materials and Methods

### 2.1. Chemicals

All the chemicals used were of analytical grade and ≥99.9% pure. Methanol and 85% *w*/*w* phosphoric acid were purchased from Sigma-Aldrich (Steinheim, Germany). LC-MS-grade acetonitrile, formic acid, and water were purchased from Merck (Darmastadt, Germany). Standards of citric acid, fumaric acid, tryptophan, homovanillic acid, caffeic acid (CAc), syringic acid, ferulic acid, luteolin-7-*O*-glucoside, rosmarinic acid (RAc), luteolin, isorhamnetin, apigenin, and hispidulin were purchased from Extrasynthese (Genay Cedex, France) and TransMIT (Giessen, Germany). Ultrapure water (18 MΩ·cm) was obtained with a Milli-Q Advantage A10 System (Millipore, Milan, Italy). The Folin–Ciocalteu reagent was purchased from Sigma-Aldrich (St. Louis, MO, USA).

### 2.2. Plant Material and Sample Preparation

**Figure 1:**
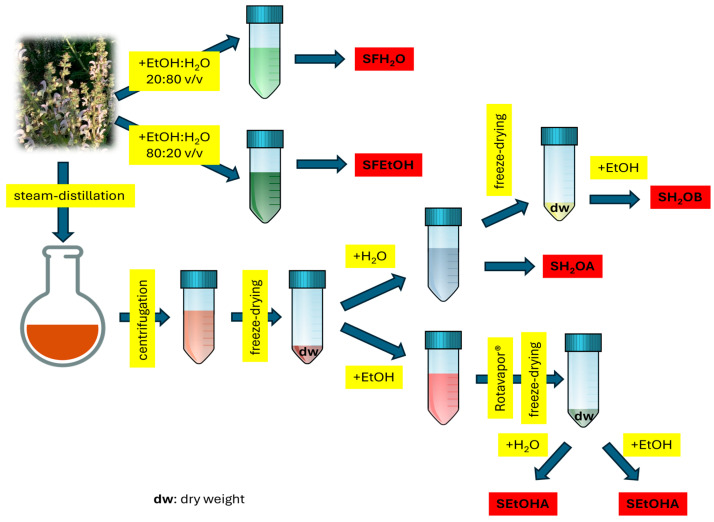
Extraction procedure and samples used for the investigation (red box).

*S. desoleana* flowering tops were collected in July 2024 with a random-block design sampling by professional pickers from plants growing under environmental conditions in Baratili San Pietro (OR, Sardinia, Italy). The specimens were identified by Prof. Cinzia Sanna (University of Cagliari, Italy), and a voucher sample (number DISVA.ALI.02.2024) was deposited at the Department of Life and Environmental Sciences of the University of Cagliari (Italy). The fresh flowering tops (15 kg) were subjected to steam distillation to extract the essential oil using an Albrigi Luigi (Stallavena, VR, Italy) 125 L stainless steel apparatus for 2 h and 30 min distillation time. The steam was generated from 20 L of water placed at the bottom of the apparatus. Aqueous fractions residual from the process were collected, centrifuged for 15 min at 4000 rpm at 10 °C, stored at −80 °C, and then freeze-dried (LIO 5Pascal freeze dryer, Trezzano sul Naviglio, Italy). Figure 1 reports the extraction procedure of the water by-product and the samples used for the investigation. The freeze-dried residue was extracted using two solvents: water and ethanol 96% (1 g of freeze-dried matrix was used with 40 mL of solvent). An aliquot of the water extract was separated (sample SH_2_OA), and the rest was freeze-dried and solubilised with ethanol 96% (sample SH_2_OB). The ethanol 96% extract was concentrated under vacuum (t = 40 ± 2 °C, Büchi Rotavapor R-114, Flawil, Switzerland) and freeze-dried: the dry residue was solubilised with either water (sample SEtOHA) or ethanol 96% (sample SEtOHB).

An aliquot of fresh flowering tops was extracted with a mixture of either ethanol 96%:water 80:20 *v*/*v* or ethanol 96%:water 20:80 *v*/*v*, with a ratio of 1:5 plant–solvent mixture. The extraction was first performed with the help of an Utraturrax^®^ (IKA-Werke, Staufen, Germany) at room temperature, and then using ultrasonification at 10 °C for 30 min. The extract was centrifuged for 15 min, and the supernatant was separated and analysed by LC-MS and LC-HPLC (samples SFEtOH and SFH_2_O).

### 2.3. High-Resolution LC-ESI-QTOF-MS/MS and HPLC-DAD Analyses

The *S. desoleana* steam-distillation water by-product was evaluated qualitatively and quantitatively using the approach provided by De Luca et al. [21]. The experiments used an electrospray ionisation (ESI) source in both positive and negative ion modes. To summarise, the analytical setup included an advanced ion mobility QToF LC/MS system, a 1290 Infinity II UPLC, and a 6560 IM-QTOF (Agilent Technologies Inc., Palo Alto, CA, USA). Data acquisition and processing were done using Agilent MassHunter Workstation Acquisition software v. B.09.00. (Agilent Technologies). ESI/QTOF MS data were then analysed using the molecular feature extraction algorithm of the MassHunter Workstation Qualitative Analysis software v. 10.0 (Agilent Technologies). The MassHunter METLIN metabolite PCDLdatabase B.08.00 (Agilent Technologies), comparison between the experimental MS/MS spectra and spectra from a publicly available mass spectral data repository (e.g., Sirius^®^ software v. 5.5.5 [22,23] and MZmine^®^ v. 4.3 [24]), and fragmentation patterns that have been published in the literature and other natural products databases (KNApSAcK^®^, PubChem^®^, Coconut^®^) were used for the tentative identification of the metabolites and predict fragmentation and molecular formulae. Phenolic substances were quantitatively analysed using a 1260 Infinity II HPLC system and an Agilent Technologies G4212B photodiode array detector. To process the chromatograms and spectra, OpenLab CDS software version 2.51 (Agilent Technologies) was utilised. The absorption of the compounds at specific wavelengths (flavonoids at 360 nm, hydroxycinnamic acids at 313 nm, hydroxybenzoic acids at 280 nm, tryptophan, and homovanillic acid at 210 nm) was measured to identify and quantify them. The calibration curves were built using the least squares approach to correlate the peak area with the concentration, with R^2^ > 0.999 for all standards in the 0.2–10.0 mg/L range. For the quantitative analysis, plant extracts were initially solubilised with an EtOH:H_2_O 80:20 *v*/*v* mixture (plant extract–solvent ratio 1:50 *w*/*v*) and diluted 1:1–1:20 *v*/*v* with 0.22 M H_3_PO_4_. Prior to injection, the solutions were filtered using a 0.22 μm CA syringe filter. The compound amount was expressed as mg/g dw (dry weight).

### 2.4. Determination of Total Polyphenols, Antioxidant Capacity, and Reducing Power via Spectrophotometric Assays

All experiments were conducted on a Cary 50 spectrophotometer (Varian, Leinì, TO, Italy) using 10 mm Kartell^®^ plastic cuvettes. Before the analysis, extract samples were suitably diluted with MeOH in the 1:5–1:100 *v*/*v* range to fit the calibration curve ranges. The TP content was determined using the modified Folin–Ciocalteu spectrophotometric technique [25,26]. In brief, the absorbance was measured with respect to a blank at 725 nm and, utilising a calibration curve of a newly made gallic acid standard solution (10–200 mg/L), the TP results were expressed as milligrams of gallic acid equivalent (GAE) per gram of dw. The DPPH^•^ assay was conducted in compliance with Tuberoso et al. [25]: 50 μL of diluted extract or standard was added to 10 mm cuvettes along with 2000 μL of DPPH^•^ solution (0.04 mmol/L in methanol). The spectrophotometric measurements were made at 517 nm after 60 min. The ABTS^•+^ assays were conducted in line with Bouzabata et al. [26] and Re et al. [27]: 10 mm cuvettes were filled with 20 μL of sample (diluted extract or standard or methanol as blank) and 2000 μL of 0.08 mM ABTS^•+^ solution. The mixture was then swirled, and spectrophotometric measurements were made at 734 nm. The quantitative measurement of the DPPH^•^ and ABTS^•+^ assays was performed using Trolox as an external standard (0.2–1.0 mmol/L) and expressing data as mmol TEAC (Trolox equivalent antioxidant capacity)/g of dw. To analyse the FRAP assay, a ferric complex of 2,4,6-tris(pyridin-2-yl)-1,3,5-triazine (TPTZ) and Fe^3+^ was created, in accordance with Bouzabata et al. [26]. In 10 mm cuvettes, 20 μL of the standard or the diluted extract solution was additionally mixed with 2000 μL of freshly prepared reagent (0.3123 g TPTZ and 0.5406 g FeCl_3_·6H_2_O in 100 mL of acetate buffer pH = 3.6). The spectrophotometric measurements at λ = 593 nm were made after 60 min. With a few minor modifications, the CUPRAC test was conducted in compliance with Bouzabata et al. [26] and Bektaşoğlu et al. [28]. Moreover, 10 mm polystyrene cuvettes were filled with 1000 μL of water, 500 μL of copper (II) chloride, 500 μL of neocuproine, 500 μL of ammonium acetate, and 100 μL of methanol (blank), standard, or sample, in that order. Spectrophotometric measurements were made at λ = 450 nm after 30 min. The FRAP and CUPRAC assays were quantitatively analysed using the external standard approach, and results were reported as mmol Fe^2+^/g of dw using ferrous sulphate in the 0.1–2 mmol range.

### 2.5. Cell Culture Maintenance

The Caco-2 cell line was obtained from ECACC (Salisbury, UK). Caco-2 cells come from human colorectal adenocarcinoma, which, once reaching confluence, differentiate into normal enterocytes. Dulbecco’s modified Eagle’s medium (DMEM) with low glucose and with L-arginine, phosphate-buffered saline (PBS) without MgCl_2_ and CaCl_2_, foetal bovine serum (FBS), and penicillin/streptomycin 1X were obtained from Euroclone (Milan, Italy). Caco-2 cells were grown in T75 flasks until their confluence reached 80% at 37 °C in a 5% CO_2_ humidified atmosphere in DMEM supplemented with 10% FBS and 1% antibiotic/antimycotic solution (100 U/mL penicillin, 100 mg/mL streptomycin) [29]. At passage 25–40, cells were removed from flasks by adding a trypsin solution at 1% and incubating at 37 °C for 5–10 min. Caco-2 cells were then collected and centrifuged, and then seeded into 6- or 96-well plates at a concentration of 5 × 10^4^ cells/mL for different experiments. Cells were cultured by replacing the medium twice weekly.

### 2.6. MTT Viability Test

To determine any cytotoxic activity of the phenolic extracts in normal differentiated (21 days post-seeding) cells, Caco-2 cell viability was assessed using an MTT assay [30]. The cells were seeded in 96-well plates (5 × 10^3^ cells/well in 100 μL) and incubated for 24 h with different concentrations (5–250 μg/mL) of methanolic dilutions of SEtOHA and SH_2_OA extracts or with an equivalent volume of methanol for the Controls. At 24 h before treatment, the 10% serum-supplemented medium was discarded and replaced with a medium with 2.5% serum. After incubation, the medium was removed, and 100 μL of the MTT solution (5 mg/mL of MTT in PBS, 8%, in fresh serum-free medium, 92%) was added and left for 4 h at 37 °C. The MTT solution was then removed, and 100 μL of DMSO was added to each well. Subsequently, the absorbance of each well was measured at 570 nm using a microplate reader (Infinite F200, Tecan, Salzburg, Austria). Sixteen independent assays were performed, and cell survival was expressed as a percentage of Control (0 μg/mL) values.

### 2.7. Determination of Intracellular ROS Production

ROS production in Caco-2 cells was evaluated using the fluorescent probe H_2_-DCF-DA as reported by Gil et al. [31], with slight modifications. Cells were seeded in 96-well plates and, once differentiated, were incubated with 10 μM of H_2_-DCF-DA in 100 μL of PBS for 30 min. Subsequently, H_2_-DCF-DA was replaced by the PBS solution containing the SEtOHA and SH_2_OA extracts (5, 10, 25, 50 and 100 μg/mL) 30 min before adding tert-butyl hydroperoxide (TBH) 2.5 mM to induce ROS release and lipid peroxidation. Control cells were treated only with PBS. The increase in cell fluorescence was determined using an Infinite F200 (Tecan, Salzburg, Austria) microplate reader at 485 and 530 nm (excitation and emission wavelengths, respectively). ROS production was monitored by reading the fluorescence emitted every five minutes and after 120 min of incubation in eighteen independent experiments.

### 2.8. Determination of Reduced and Oxidised Intracellular Glutathione and Cystein

Intracellular reduced glutathione (GSH), cysteine (CyS), oxidised glutathione (GSSG), and cystine (CySS) levels were determined by high-performance liquid chromatography coupled to an electrochemical detector (HPLC-EC), as previously described [32,33]. In detail, Caco-2 cells were seeded in 6-well multiwells at a density of 1 × 10^5^ cells/2 mL and incubated for 14–21 days to achieve complete differentiation. Cells were then treated with the extract at 10, 25, and 50 μg/mL against the prooxidant activity led by TBH (final concentration 2.5 mM) for 30 min. After incubation, cells were detached from the wells with 500 μL of PBS and centrifuged for 7 min at 12,500 rpm. The precipitate was then extracted with 150 μL of 10% metaphosphoric acid (MPA) and 150 μL of 0.05% trifluoroacetic acid (TFA) (Merck, Milan, Italy). After centrifugation, the supernatant was collected and injected into the HPLC system. The amounts of GSH, CyS, GSSG, and CySS were measured using an HPLC (Agilent 1260 infinity, Agilent Technologies) equipped with an electrochemical detector (DECADE II Antec, Leiden, The Netherlands) and an Agilent 35900E interface. Data were collected from three independent experiments and expressed as a percentage of Control cells (100%) of the ratio GSH/GSSG and Cys/CySS.

### 2.9. Determination of NO Release

To assess NO production, the Griess reagent method was used to quantify NO metabolites (nitrites) as previously reported [34]. Briefly, Caco-2 cells were cultured in 6-well plates (5 × 10^4^ cells/mL in 2 mL) in phenol red-free DMEM (Euroclone, Milan, Italy) with 0.1 mM L-arginine and 2.5% foetal bovine serum. NO release was measured by incubating the cells for 48 h with the extract at concentrations of 10, 25 and 50 μg/mL, together with lipopolysaccharide (LPS, 1 μg/mL) as a pro-inflammatory stimulus. After incubation, the medium was aliquoted from the wells, and the nitrite concentration was determined by mixing 100 μL of the same medium with an equal volume of Griess’ Reagent (Sigma Aldrich, Milan, Italy) and incubating for 20 min at room temperature. Subsequently, the samples were analysed spectrophotometrically at 540 nm, and nitrite levels were determined with a sodium nitrite standard curve ranging from 0.1 to 10 μM. Sixteen independent assays were performed, and the results were expressed as μmol/L of nitrite secreted by the cells.

### 2.10. In Silico Studies

The crystal structures of inducible nitric-oxide synthase (iNOS, PDB 4NOS [35]), the catalytic domain of NADPH oxidase 2 (NOX2, 2CDU [36]) and the Kelch repeat domain of Keap1 bound to the Nrf2 Neh2 peptide (4L7B [37]) were downloaded from the Protein Data Bank and prepared with Schrödinger’s Protein Preparation Workflow (v 2024-4): bond orders were assigned, missing atoms and side chains added, hydrogen-bond networks optimised, and heavy atoms minimised with the OPLS4 force-field at pH 7.4 [38,39,40]. RAc, methyl rosmarinate (MR), CAc, and luteolin-7-*O*-glucuronide (LG) were built with LigPrep, generating up to ten low-energy conformers per ligand after Epik protonation at pH 7.4 ± 0.5 [41]. Flexible docking was then performed in Glide Extra-Precision mode with “Expanded” pose sampling; 20 × 20 × 20 Å grids were centred on the co-crystallised ligands to encompass the full binding pockets [42]. Validation by ligand redocking reproduced the experimental poses with RMSD < 1.1 Å and favourable XP scores (iNOS: 0.251 Å, −12.841 kcal/mol; NOX2: 1.057 Å, −13.280 kcal/mol; Keap1: 0.395 Å, −7.835 kcal/mol), confirming the robustness of the computational protocol.

### 2.11. Statistical Analyses

Data were analysed by means of the software GraphPad Prism 5 (GraphPad software, San Diego, CA, USA) using one-way analysis of variance (ANOVA) followed by the Bonferroni post hoc test. A level of *p* < 0.05 was considered statistically significant.

## 3. Results and Discussion

The water residue from the *S. desoleana* steam distillation was dehydrated, and the extract was dissolved with water and/or ethanol 96% to verify if it was possible to obtain extracts with improved antioxidant capacities. The four samples obtained (SH_2_OA, SH_2_OB, SEtOHA, and SEtOHB; Figure 1) were investigated for their composition in polar compounds and antioxidant activities. Moreover, two samples obtained from the fresh flowering tops (SFEtOH and SFH_2_O) were used to verify if the compounds detected in the water residue were originally present in the plant or were produced during the steam-distillation procedure (Table S1).

### 3.1. Qualitative Determination of Bioactive Compounds in S. desoleana Steam-Distillation Water Residue

(HR) LC-ESI-QTOF MS/MS was used to qualitatively analyse the extract in both the positive and negative ion modes (Table S1), and HPLC-PDA analysis was used to quantify targeted chemicals. The two most representative extracts were analysed and compared with the extracts of the fresh flowering tops. By comparing the experimental MS/MS spectra with the fragmentation patterns reported in the literature or with the fragmentation patterns and spectra reported in a public repository of mass spectral data, the 28 distinct compounds were identified (Figure S1). Other methods of identification included comparing the *m*/*z* values with those reported in the literature [23]. According to their LC-PDA retention times, the compounds found in the by-product extracts are listed in Table S1, which also includes the molecular formula obtained by mass measurement (experimental result), MS/MS results, mass error (∆ppm), identification references, and identification confidence levels [43].

The 28 compounds detected in the *S. desoleana* steam-distillation water by-products were mainly classified as glycosyl and glucuronic derivatives of flavonoid compounds, organic acids, coumaric acids, amino acids, and catechols. Compound **1** was identified as citric acid due to the [M−H]^−^ at *m*/*z* 191.0201 and fragments at 87.0091 and 111.0085 and due to the [M+H]^+^ at *m*/*z* 193.0456, as well as through comparison with literature data on other *Salvia* species [44,45]. Compound **2** was identified as fumaric acid due to the [M−H]^−^ at *m*/*z* 115.0701 and due to the [M+H]^+^ at *m*/*z* 117.0583, as well as through the comparison with pure standard, and it was detected in previous studies on *S. poculata* and *S. officinalis* [46,47]. Compound **3** was tentatively identified as protocatechuic acid hexoside due to the [M−H]^−^ at *m*/*z* 315.0731 and fragments at 152.0106 and 108.0211, as well as the comparison with previous literature data [48]. Peak 4 was tentatively attributed to salvianic acid A (danshensu) due to the [M−H]^−^ at *m*/*z* 197.1700 with fragments at 135.0446 and 123.0447 and due to the [M+H]^+^ at *m*/*z* 221.0419 (adduct with Na^+^), as well as the comparison with literature data [49]. Peak 5 was attributed to tryptophan due to the [M−H]^−^ at *m*/*z* 203.0827 and the [M+H]^+^ at *m*/*z* 227.0793 (adduct with Na^+^) with fragments at *m*/*z* 146.0602 and 118.0646, as well as the comparison with pure standard. Tryptophan was previously identified in other plants from the *Lamiaceae* family, such as *S. miltiorrhiza* [50]. Compound **6** was identified as homovanillic acid (C_9_H_10_O_4_) due to the [M−H]^−^ at *m*/*z* 181.0879 and a fragment at 135.0445 and the [M+H]^+^ at *m*/*z* 183.0976, and it was previously found in *S. elegans* [51]. Peak 7 was attributed to CAc with a molecular formula C_9_H_8_O_4_ due to the [M−H]^−^ at *m*/*z* 179.0367 with fragments at *m*/*z* 179.0300 and 135.0400 and the [M+H]^+^ at *m*/*z* 181.0455, as well as the comparison with literature data [52,53,54,55]. Compound **8** was tentatively identified as tuberonic acid glucoside due to the [M−H]^−^ at *m*/*z* 387.1709 with fragments at *m*/*z* 207.1000 and 59.0100 and the [M+H]^+^ at *m*/*z* 411.1628 (adduct with Na^+^) with fragments at *m*/*z* 85.0634, 209.1199 and 191.1069, as well as the comparison with previous studies on *S. sclarea* [55]. Compound **9** was tentatively identified as salvianic acid C due to the [M−H]^−^ at *m*/*z* 377.9070 with fragments at *m*/*z* 161.0231 and 179.0227, as well as the comparison with previous studies on other *Salvia* species [45,56]. Peak 10 was identified as syringic acid due to the [M−H]^−^ at *m*/*z* 197.0459 with fragments at *m*/*z* 182.0205 and 138.0321 and the [M+H]^+^ at *m*/*z* 199.0598, as well as the comparison with pure standard and literature data on other species [57,58]. Peak 10 was identified as ferulic acid due to the [M−H]^−^ at *m*/*z* 193.0511 with fragments at *m*/*z* 134.0358 and 89.0397 and the [M+H]^+^ at *m*/*z* 195.0649, as well as the comparison with pure standard and literature data on other species [45]. Compound **12** was identified as luteolin 7-*O*-glucoside due to the [M−H]^−^ at *m*/*z* 447.0946 with fragments at *m*/*z* 285.0410 (loss of a luteolin unit) and 284.0330 and the [M+H]^+^ at *m*/*z* 449.1089 with a fragment at 287.0560, as well as the comparison with pure standard and previous studies on other *Salvia* species [52,55,59]. Peak 13 was tentatively attributed to LG due to the [M−H]^−^ at *m*/*z* 461.0967 with a fragment at *m*/*z* 285.0410 (loss of a luteolin unit) and the [M+H]^+^ at *m*/*z* 463.0876 with a fragment at *m*/*z* 287.0553, as well as the comparison with previous studies on *S. spinosa* and *S. palestina* [59]. Compound **14** was identified as isorhamnetin hexoside due to the [M−H]^−^ at *m*/*z* 477.1062 with fragments at *m*/*z* 315.0502 (loss of a isorhamnetin unit) and 299.0208 and the [M+H]^+^ at *m*/*z* 501.1002 (adduct with Na^+^), as well as the comparison with previous studies on other *Salvia* species [45,56]. Peak 15 was identified as apigenin hexoside due to the [M−H]^−^ at *m*/*z* 431.1002 with fragments at *m*/*z* 269.0440 (loss of an apigenin unit) and 268.0374 and the [M+H]^+^ at *m*/*z* 433.1138 with a fragment at 271.0604, as well as the comparison with previous studies [45,59]. Compound **16** was identified as RAc due to the [M−H]^−^ at *m*/*z* 359.0780 with fragments at *m*/*z* 161.0241, 197.0449 and 179.0344 and the [M+H]^+^ at *m*/*z* 383.0737 (adduct with Na^+^) with fragments at *m*/*z* 163.0387 and 135.0446, as well as the comparison with pure standard and previous studies on several *Salvia* species [52,53,54,55,57,58,59]. Compound **17** was tentatively identified as apigenin glucuronide with molecular formula C_21_H_18_O_11_ due to the [M−H]^−^ at *m*/*z* 445.8967 with fragments at *m*/*z* 269.0457 (loss of an apigenin unit) and the [M+H]^+^ at *m*/*z* 447.0936 with a fragment at *m*/*z* 271.0610, as well as the comparison with literature data [59]. Peak 18 was tentatively attributed to hispidulin glucuronide due to the [M−H]^−^ at *m*/*z* 475.3450 with molecular formula C_22_H_22_O_11_ and with fragments at 299.0563 (loss of a hispidulin unit) and 284.0328 and the [M+H]^+^ at *m*/*z* 477.1044 with fragments at *m*/*z* 301.0710, as well as the comparison with previous studies on other *Salvia* species [59]. Compound **19** was tentatively identified as salvianolic acid K with molecular formula C_27_H_24_O_16_ due to the [M−H]^−^ at *m*/*z* 555.1161 with fragments at *m*/*z* 161.0243 and 135.0449 and the [M+H]^+^ at *m*/*z* 579.1103 (adduct with Na^+^), as well as the comparison with literature data [44,55]. Compound **20** was tentatively identified as methyl rosmarinate with molecular formula C_19_H_18_O_8_ due to the [M−H]^−^ at *m*/*z* 373.2098 with fragments at *m*/*z* 135.0455 and 175.0405 and the [M+H]^+^ at *m*/*z* 397.0894 (adduct with Na^+^) and 145.0239 and 117.0324, as well as the comparison with literature data [44,54]. Peak 21 was attributed to luteolin aglycone due to the [M−H]^−^ at *m*/*z* 285.0418 and the [M+H]^+^ at *m*/*z* 287.0560, as well as the comparison with pure standard and literature data [53,55,57,58]. Peak 22 was attributed to isorhamnetin aglycone due to the [M−H]^−^ at *m*/*z* 315.0978 with a fragment at *m*/*z* 300.0283 and the [M+H]^+^ at *m*/*z* 317.0663 with a fragment at *m*/*z* 302.0426, as well as the comparison with pure standard and literature data [44,60]. Compound **23** was tentatively attributed to apigenin acetyl-glucoside with molecular formula C_23_H_22_O_11_ due to [M−H]^-^ at *m*/*z* 473.1103 and fragments at 269.0454 (loss of an apigenin unit) and 268.0370 and due to the [M+H]^+^ at *m*/*z* 475.1234 with a fragment at 271.0605, as well as the comparison with previous studies on *S. officinalis* [61]. Peak 24 was attributed to apigenin aglycone due to the [M−H]^−^ at *m*/*z* 269.0468 and the [M+H]^+^ at *m*/*z* 271.0612, as well as the comparison with pure standard and literature data [52,53,55,58]. Compound **25** was tentatively identified as dimethyl-quercetin ether with molecular formula C_17_H_14_O_7_ due to the [M−H]^−^ at *m*/*z* 329.0683 with a fragment at *m*/*z* 299.0207 and 314.0453 and the [M+H]^+^ at *m*/*z* 331.0818 with fragments at 298.0472 and 316.0573, as well as the comparison with literature data [48,56]. Compound **26** was identified as hispidulin aglycone due to the [M−H]^−^ at *m*/*z* 299.0572 with a fragment at *m*/*z* 284.0319 and the [M+H]^+^ at *m*/*z* 301.0709 with a fragment at *m*/*z* 286.0471, as well as the comparison with pure standard and literature data on *S. fruticose* [62]. Compound **27** was tentatively identified as crisimaritin with molecular formula C_17_H_14_O_6_ due to the [M−H]^−^ at *m*/*z* 313.0987 with a fragment at *m*/*z* 283.0251 and the [M+H]^+^ at m/z 315.0886 with fragments at *m*/*z* 282.0536 and 300.0620, as well as the comparison with literature data [44,59]. Peak 28 was tentatively attributed to genkwanin with molecular formula C_16_H_12_O_5_ due to the [M−H]^-^ at *m*/*z* 283.0622 and a fragment at 268.0387 and due to the [M+H]^+^ at *m*/*z* 285.0765, as well as the comparison with literature data [45].

### 3.2. Quantitative Determination of Bioactive Compounds in S. desoleana Steam-Distillation Water Residue

HPLC-PDA analysis was used to quantify target compounds in the four samples obtained from the *S. desoleana* steam-distillation water residue. Table 1 reports the amount of the single compounds (mg/g dw) and the total quantity for the main classes (flavonoids, hydroxycinnamic acids, hydroxybenzoic acids, and other compounds). Accounting for the total phenols dosed by LC-PDA, it can be noticed that sample SEtOHA is the richest in bioactive compounds, followed by SH_2_OA, SEtOHB and finally SH_2_OB (553.82 ± 7.47, 399.64 ± 17.36, 205.93 ± 0.59, 27.07 ± 0.28 mg/g dw, respectively). Hydroxycinnamic acids are the most representative compounds, with RAc the most abundant in all samples. The highest amount of RAc is in SEtOHA (427.42 ± 1.99 mg/g dw). RAc was previously studied for its resistance to high temperature [63], and this could justify its predominant presence in our extract. RAc, a naturally occurring phenolic compound found in plants in the *Lamiaceae* family, has received a lot of interest due to its wide range of biological activity and medicinal potential. For instance, it has AA, anti-inflammatory properties, neuroprotective effects, antidiabetic and antimicrobial activity, cardioprotective effects, and hepatoprotective activity [64,65], and, thanks to its antioxidant properties, it can modulate cancer-related pathways [66]. Among hydroxycinnamic acids, MR, a RAc derivative, is another notable compound, still the most abundant in SEtOHA (17.42 ± 1.03 mg/g dw). Beyond its antioxidant properties [67], MR inhibits different enzymes belonging to important metabolic pathways, such as lipoxygenase, angiotensin-converting enzyme (ACE) [67], and tyrosinase [68].

Another notable compound is CAc, which is once again the most abundant in SEtOHA (26.69 ± 0.49 mg/g dw), followed by SH_2_OA, SEtOHB, and SH_2_OB (15.33 ± 0.19, 11.86 ± 0.00, and 1.19 ± 0.01 mg/g dw, respectively). CAc is a noteworthy compound, known for its hepatoprotective activity, antimicrobial activity, antidiabetic activity, and AA [69]. Following hydroxycinnamic acids, flavonoids are the second most characteristic class of compounds. Interestingly, total flavonoids are higher in SH_2_OA than in SEtOHA, contrasting with the global trend for the other classes of compounds. Among flavonoids, the most representative one is LG (11.04 ± 0.10 mg/g dw), followed by hispidulin glucuronide, apigenin glucuronide, and luteolin 7-*O*-glucoside (5.30 ± 0.17, 5.05 ± 0.05 and 3.08 ± 0.16 mg/g dw). Primarily, the most abundant flavonoids are conjugated with glucuronic acid. LG is well known for its anti-inflammatory and antioxidative properties, for instance, inhibiting cyclooxygenase-2, interleukin-6, and tumour necrosis factor (TNF)-α [70]. Among hydroxybenzoic acids, syringic acid is the most abundant, and, notably, it has the highest value in SEtOHB (0.62 ± 0.00 mg/g dw). It is followed by SEtOHB and SH_2_OA at the same value (0.44 ± 0.21 and 0.44 ± 0.03 mg/g dw, respectively), and is not determined in SH_2_OB. Interestingly, it can be noticed that there are some degradation products of the salvianolic acids, such as salvianic acid A (danshensu) [71]. As far as we know, the phenolic composition of *S. desoleana* has not been investigated yet. Comparing *S. desoleana* with other *Salvia* species, it can be noticed that RAc is one of the most abundant compounds, if not the most, as can be seen in *S. clandestina*, *S. fruticosa*, *S. officinalis*, and *S. sclarea* (7.56 ± 0.51, 6.55 ± 0.18, 5.46 ± 0.31 and 15.57 ± 0.84 mg/g dry weight) [55].

### 3.3. Total Phenols and Antioxidant Activity

The four extracts obtained from the water residue were evaluated with five colourimetric assays to evaluate the total phenolic amount, the total antioxidant capacity (CUPRAC and FRAP assays), and the antiradical capacity (DPPH^•^ and ABTS^•+^ assays) (Table 2). Sample SEtOHA was the one with the highest TP content (106.02 ± 6.02 mg GAE/g dw), followed by SH_2_OA, SEtOHB, and SH_2_OB (75.28 ± 1.97, 32.44 ± 1.93, and 3.52 ± 0.22 mg GAE/g dw, respectively). Jasicka-Misiak et al. [57] reported the highest TP for the methanolic extract of *S. sclarea* (134.4 ± 9.7 and 96.1 ± 2.6 mg/g dw) and *S. officinalis* (93.8 ± 3.1 and 63.9 ± 2.9 mg/g dw), showing that *S. desolana* values are halfway between the two species. The same trend was observed for the assays used to evaluate four antioxidant and antiradical capacities, confirming SEtOHA and SH_2_OA as the two compounds with the highest capability. These extracts show overall lower values than previous comparable studies performed on *Cynara cardunculus* var. *scolymus* leaf extracts, in which, for instance, the highest values were 1.10–8.82 mmol TEAC/g dp and 3.37–31.12 mmol Fe^2+^/g dp for DPPH^•^ and FRAP, respectively, and the TP values were 198.32–1433.32 mg GAE/g dp [72].

The estimation of total phenolic amount by the Folin–Ciocalteu assay is based on a redox reaction; thus, this analysis can also be used to evaluate the antioxidant capacity. For this reason, TP values show a significant linear correlation with the antioxidant and antiradical assays (R ≥ 0.98, *p* ≤ 0.001).

### 3.4. Cell Viability and Antioxidant Activity Against TBH-Induced ROS Release

**Figure 2:**
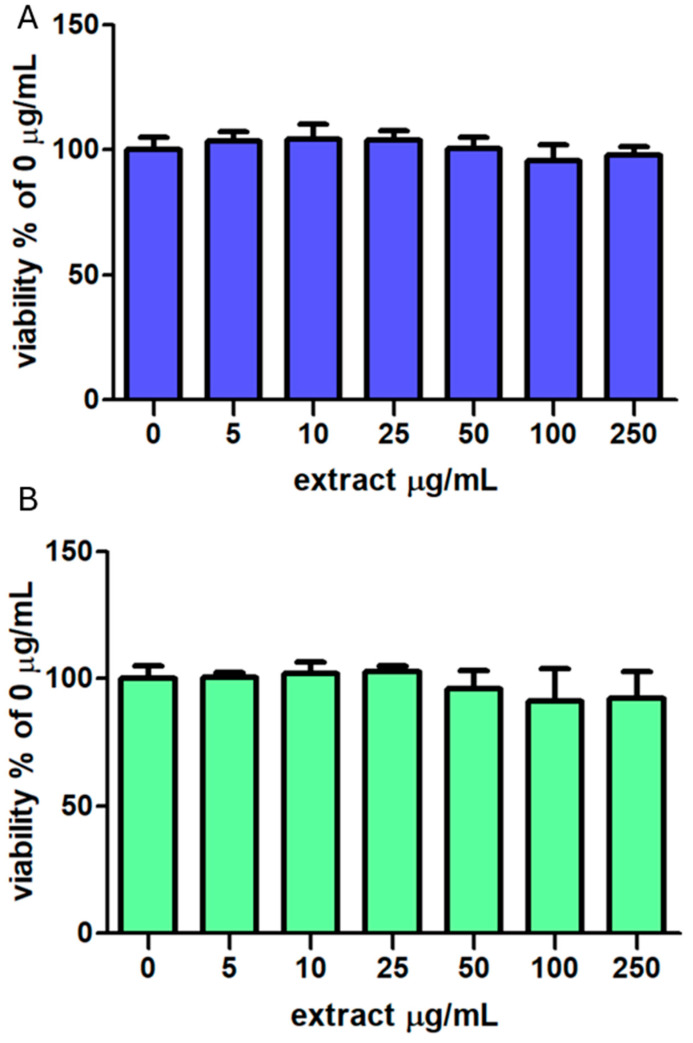
Cell viability, expressed as % of Control values (100%), measured with the MTT assay in Caco-2 cells pretreated with SEtOHA (**A**) or SH_2_OA (**B**) (5–250 μg/mL) or with an equivalent volume of MeOH (CTRL) for 24 h. Data are presented as mean ± SD (*n* = 16).

Taking into account the results reported in Table 2, on the two most active samples (SEtOHA and SH_2_OA), any eventual cytotoxic effect on differentiated Caco-2 cell monolayers and the potential antioxidant effects against TBH-induced ROS production were investigated. Firstly, cell viability through the MTT assay was evaluated, and the data showed no toxic effects at all concentrations tested, and no significant statistical results were observed (*p* > 0.05, Figure 2A,B).

**Figure 3:**
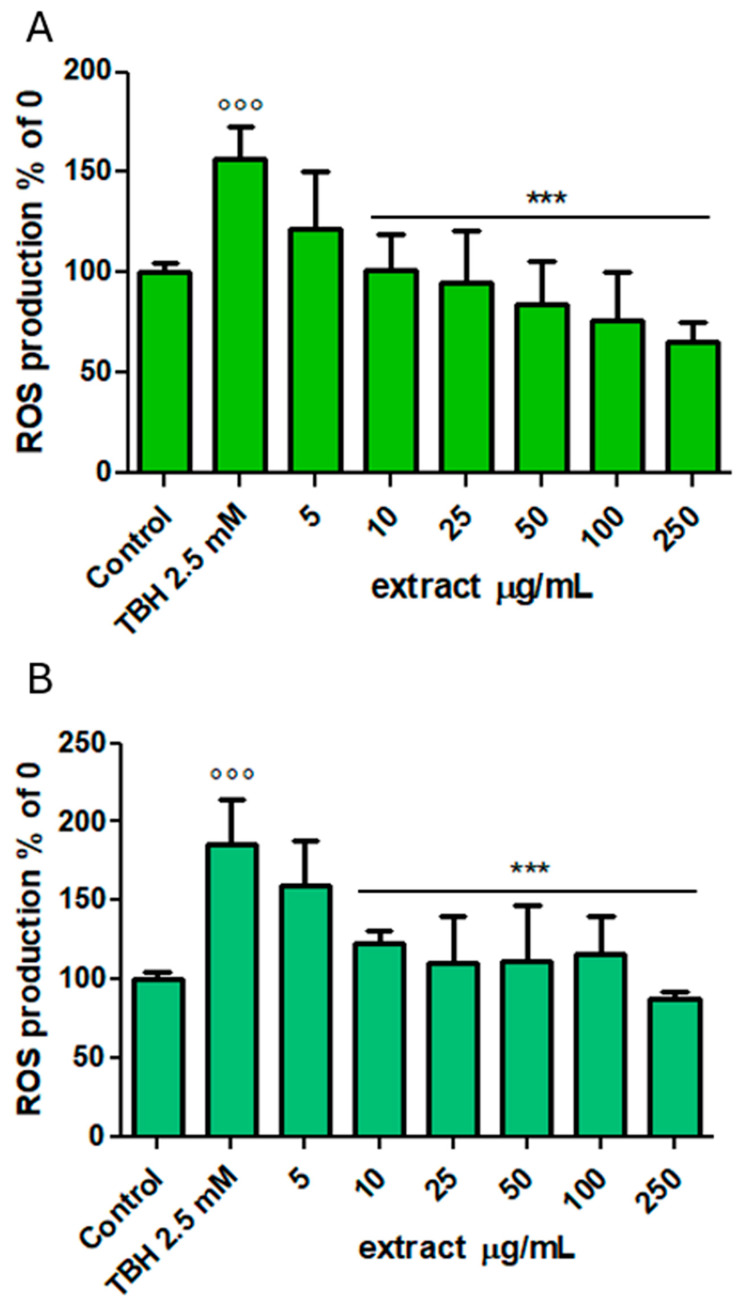
ROS levels, shown as H_2_-DCF-DA fluorescence and expressed as % of the Control samples (CTRL, non-oxidised nor pretreated samples) in differentiated Caco-2 cells after 2 h incubation with SEtOHA (**A**) or SH_2_OA (**B**) (5–250 μg/mL) in co-incubation with TBH (2.5 mM). *** = *p* < 0.001 vs. TBH 2.5 mM, °°° = *p* < 0.001 vs. Control (*n* = 18).

An experiment was then conducted at non-toxic concentrations to evaluate the protective action of the phenolic extracts (Figure 3). The antioxidant capacity was evaluated in differentiated Caco-2 cells exposed to the oxidising action of TBH (2–5 mM).

After 120 min of incubation, TBH determined in the treated cells a significant production of intracellular ROS, with levels approximately twofold lower with respect to the untreated cells (*p* < 0.001 vs. Control). Pretreatment with both the phenolic extracts, starting from 10 μg/mL, counteracted the TBH-induced alteration of cellular redox status, with a significant and dose-dependent decrease in ROS level (*p* < 0.001 vs. TBH 2.5 mM). Indeed, the most interesting results were observed at the highest concentration tested of 250 μg/mL, confirming the positive impact of phenolic compounds on oxidative stress in the intestinal environment. These results confirm what has already been observed in HUVEC endothelial cells treated with supercritical carbon dioxide phenolic extracts at concentrations even lower than those tested in this study (0.1, 1 and 10 μg/mL), which showed significant AA against hydrogen peroxide [73]. Furthermore, it can be seen from Figure 3 that both SEtOHA and SH_2_OA extracts showed a superimposable activity, without presenting differences with regard to the dose-dependent activity belonging to their phenolic profile.

### 3.5. Intracellular Antioxidant Levels

**Figure 4:**
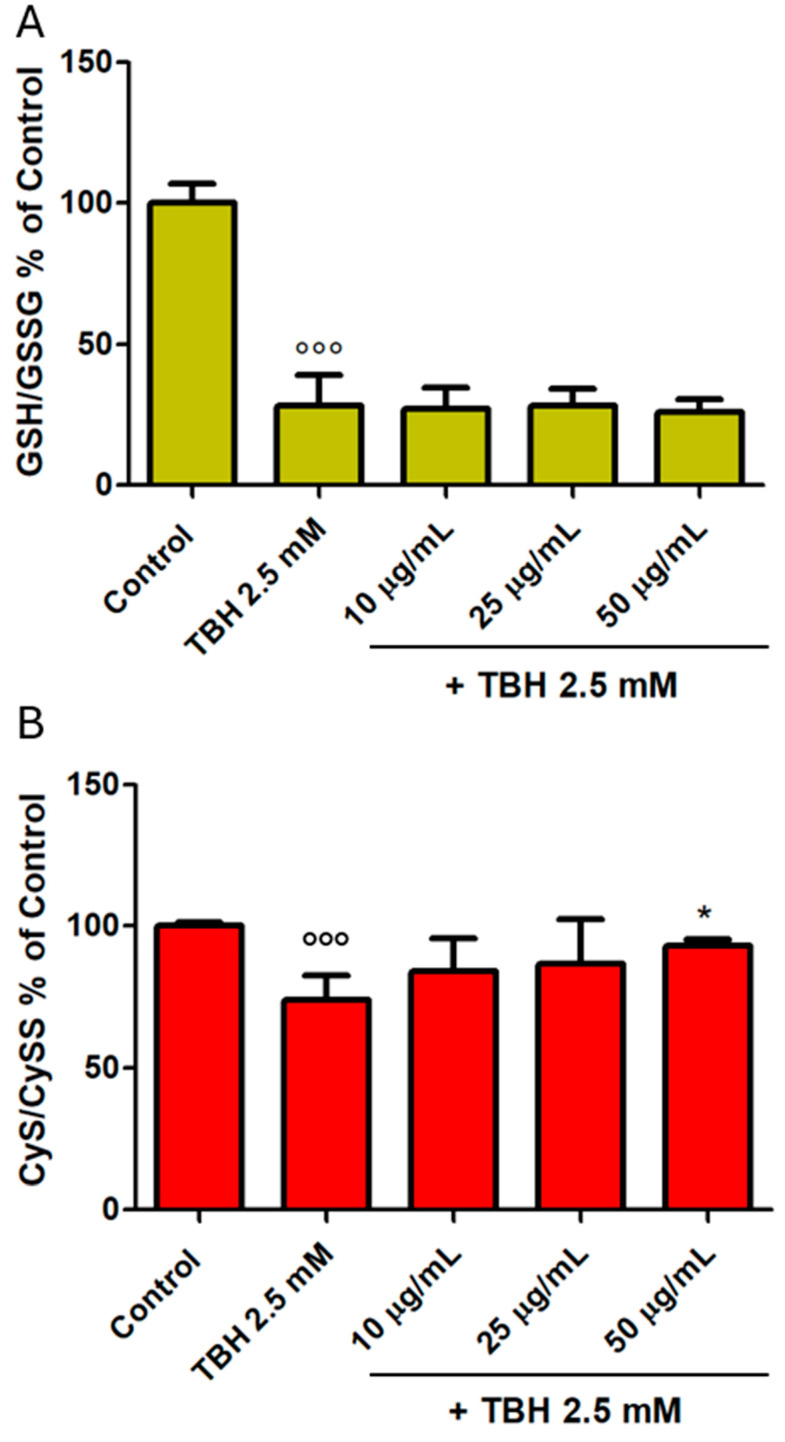
Levels of GSH/GSSG ratio (**A**) and levels of CyS/CySS ratio (**B**) after 30 min of incubation with SEtOHA (10–50 μg/mL) in co-incubation with TBH (2.5 mM). Levels of intracellular aminothiols were expressed as the ratio of reduced and oxidised forms in percentage with respect to Control values (100%). Data are expressed as mean ± SD. All experiments were performed three times independently, each time in triplicate, to confirm the results. °°° = *p* < 0.001 vs. Control; * = *p* < 0.05 vs. TBH 2.5 mM.

GSH is one of the most representative intracellular antioxidants, and its ratio with GSSG may be used as a marker of oxidative stress. Given the same effectiveness against the oxidising action of TBH, as shown in Figure 3, we chose to test only the SEtOHA extract because it could be potentially more biologically active, given its higher content of TP, as highlighted in Table 2. In addition, in this experimental model, we tested the lowest concentrations found to be active against oxidative stress in the previous preliminary test (10–50 µg/mL, Section 3.4) since it was seen that biological activity occurred within this lower range, making higher concentrations unnecessary. Moreover, higher concentrations may exceed physiologically relevant or therapeutically achievable levels. In our experiments, TBH led its prooxidant effects, inducing a significant decrease in the GSH/GSSG ratio (*p* < 0.001). No protective effect of extract pretreatment (10–50 µg/mL) against TBH (2.5 mM) was instead observed after 30 min of incubation (Figure 4A; *p* > 0.05 for all concentrations tested against TBH 2.5 mM). A lower decrease in terms of percentage led by TBH was observed regarding the CyS/CySS ratio (Figure 4B), but in this instance, we observed a significant protection carried out by the extract at the highest concentration tested (50 µg/mL, *p* < 0.05), while the lowest concentrations tested did not produce any significant biological effect (*p* > 0.05).

Within cells, total GSH exists free and bound to proteins. Since the enzyme glutathione reductase, which reverts free GSH from GSSG, is constitutively active and inducible upon oxidative stress, free GSH exists almost exclusively in its reduced form. The ratio of reduced to oxidised glutathione within cells is often used to indicate cellular redox alteration [74]. Similarly, the sulphur-containing amino acid CyS can also undergo oxidation to CySS and represents a biomarker of oxidative stress [75]. Current research does not support a simple, universal preference for CyS oxidation over glutathione; instead, its oxidation is tightly regulated and context-dependent, with glutathione often acting as a buffer and redox regulator for CyS and protein thiols. Herein, only CyS was restored to its reduced form significantly by the extract compared to what was seen for GSH. In this regard, electrochemical studies reveal that CyS can be detected and reduced at more negative potentials compared to GSH, indicating a difference in their reduction behaviours. CyS reduction occurs at a lower (more negative) potential, which typically means it is more easily reduced in biological systems [76].

### 3.6. NO Release

**Figure 5:**
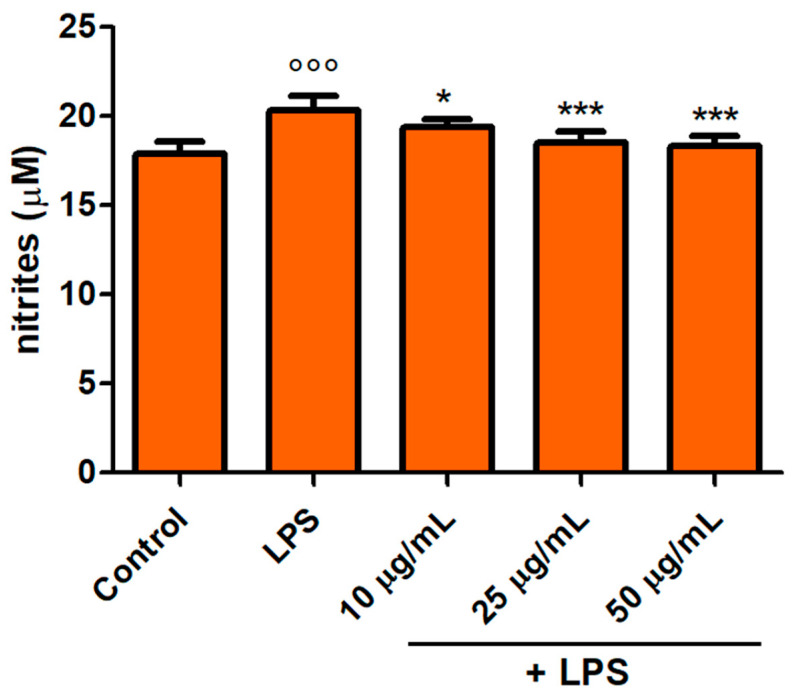
NO release (expressed as μM of nitrites) in Caco-2 cells treated with LPS (1 μg/mL) for 48 h and pretreated for 30 min with SEtOHA (10–50 μg/mL) preceding LPS co-exposure. Control and LPS groups were pretreated with an equivalent volume of methanol. Each column represents the mean ± SD of independent experiments (*n* = 16). °°° = *p* < 0.001 vs. Control; * = *p* < 0.05 vs. LPS; *** = *p* < 0.001 vs. LPS.

NO production in Caco-2 cells after 48 h LPS exposure with and without pretreatment (30 min prior to LPS co-exposure) with the SEtOHA (10–50 μg/mL) extract was then analysed. Again, lower concentrations than the initial range used in screening tests were used, as explained in Section 3.5 (10–50 μg/mL). As shown in Figure 5, LPS induced an increase (*p* < 0.001) in NO release, measured as the amount of nitrites. Contextually, all the tested concentrations of the extract significantly counteracted NO production in a dose-dependent fashion, showing excellent inhibitory activity. Anti-inflammatory effects were observed already at the lowest concentration tested (10 μg/mL, *p* < 0.05), and then increased in cells tested with the highest concentrations (25–50 μg/mL, *p* < 0.001). This analysis was performed here for the first time with regard to phenolic extracts coming from *S. desoleana*, while some similar data had been obtained in extracts of different species of *Salvia*.

Multiple types of *Salvia* extracts and their active compounds have been shown to suppress NO production, mainly by downregulating iNOS expression and interfering with key inflammatory signalling pathways in activated immune cells. In detail, most *Salvia* extracts act by downregulating iNOS expression at the transcriptional level, often through the inhibition of the NF-κB signalling pathway, while other extracts have been shown to directly scavenge NO or inhibit upstream inflammatory signals, such as Toll-like receptor 4 [77,78,79].

### 3.7. Docking and Binding-Mode Analysis

Following the results of the chemical composition and the antioxidant activities of the *S. desoleana* extracts, an in silico evaluation of target compounds (RAc, MR, CAc, and LG) on enzymes involved in the antioxidant response was performed. Namely, iNOS, Keap1-Nrf2, and NOX were selected among the enzymes potentially involved because each of them represents a specific mechanism in the control of ROS and NO release, and their biological activity is known to be affected by polyphenols. The enzymes were chosen based on their central roles in the inflammatory pathways of interest and their known involvement in the pathophysiology relevant to our study. While other enzymes, such as COX-1 and COX-2, are classic targets in inflammation research, they were not the focus here because our aim was to explore less conventional or more specific targets that could offer novel therapeutic potential or greater selectivity. Regarding NO and its biological fate, the iNOS expression has been measured in countless cell types and different experimental systems, with contrasting results depending on the cell type and the compound tested. The modulation of iNOS expression has often been studied as a function of anti-inflammatory activity, and the mechanisms involved in the biological action of polyphenols on iNOS expression and activity are multiple [80]. Numerous polyphenols can also modulate the Keap1-Nrf2 pathway, therefore not limiting themselves to inhibiting the production of ROS based on an antioxidant action. In particular, polyphenols have been shown to inhibit Keap1-Nrf2 protein–protein interaction and also induce the degradation of Keap1, thus upregulating the Nrf2-related activation and biological function [81]. Among NOXs, NOX1, NOX2, and NOX4 are the most studied isoforms, and the assessment of their actual mechanism of action is not easy [82]. Indeed, many studies have shown how most of the known effects of (poly)phenols and their metabolites on NOX1, NOX2, and NOX4 are related to the modulation of the expression of the different constituent subunits and/or posttranslational modifications involved in the assembly and translocation of the protein complexes. Conversely, scarce evidence is given on the direct action of polyphenols on NOX active sites so far.

Herein, we report the calculated binding affinities of the four target compounds (RAc, MR, CAc, and LG) toward iNOS, Keap1-Nrf2, and NOX enzymes (Table 3). RAc consistently exhibited favourable binding affinities across all tested targets, with docking scores ranging from −7.901 to −9.655 kcal/mol, aligning well with previous studies (−8.542 kcal/mol for iNOS [83]; −7.96 kcal/mol for Keap1 [84]; and −6.67 kcal/mol for NOX2 [84]). The robust binding profile is attributable to RA’s structurally polyphenolic backbone and free carboxyl group, which together enable multiple hydrogen bonds and aromatic π-π interactions.

In contrast, MR showed a significantly reduced affinity compared to RA for all targets (−6.809 kcal/mol for iNOS, −8.191 kcal/mol for Keap1, and −7.478 kcal/mol for NOX), underscoring the possible critical role of the ionisable carboxylic group in stabilising interactions with key residues in the active site.

CAc exhibited slightly lower docking scores in our study (−4.224 kcal/mol on iNOS, −5.122 kcal/mol on Keap1, and −5.412 kcal/mol on NOX) than previously reported in the literature (−7.87 kcal/mol for iNOS and −7.04 kcal/mol for NOX p67phox [85]). This indicates possible differences in methodology or the intrinsic variability of docking algorithms. However, our results are in line with a general trend reported in the literature, which describes CAc as forming smaller and weaker interactions due to its smaller size and simpler structure compared to more complex derivatives, which show higher docking affinities [85].

LG showed remarkably high docking affinity scores in our analysis (−11.649 kcal/mol on Keap1 and −11.696 kcal/mol on NOX), comparable to structurally similar flavonoid glycosides reported in the literature, such as rutin (−10.74 kcal/mol for Keap1 [85]) and verbascoside (−10.97 kcal/mol for NOX [84]). In contrast, LG showed a lower affinity towards iNOS (−4.864 kcal/mol), perhaps owing to sub-optimal orientation or steric constraints within the iNOS active site. The substantial increase in affinity observed with LG towards Keap1 and NOX highlights the role of the sugar moiety in enhancing ligand–protein interactions, presumably through additional hydrogen bonds and polar interactions that might stabilise the ligand more within the active site.

Overall, our docking study supports the hypothesis that the polyphenolic compounds of *S. desoleana* extracts, in particular RAc and LG, have considerable potential as modulators of oxidative stress-related enzymes. However, the presence of differences in binding modes across studies underlines the need for further validation through experimental binding assays and enzyme activity assays to confirm in silico predictions and to fully understand the bioactive role of these polyphenols.

The visual inspection of the top-scored poses reported in Figure S2a–c revealed recurrent interactions that rationalise the numerical ranking. As far as iNOS is concerned, RAc forms hydrogen bonds with Gln263, Tyr373, Asp382, and Val465, together with a salt bridge and π-cation interaction involving Arg381, and an additional π-cation contact with Arg388; a further hydrogen bond is predicted to the haem cofactor. MR reproduces the same network (Gln263, Tyr373, Arg381, Asp382, Arg388, Val465, haem). CAc retains the Arg381 salt bridge/π-cation pair, a hydrogen bond to Val465 and one to the haem. LG is predicted to hydrogen-bond to Tyr373, Asp382, Arg388, Ile462, Trp463, and the haem group (Figure S2a). The affinity trend appears to be governed by a balance between electrostatic anchoring and steric fit at the entrance of the heme gate. RAc benefits from two π-cation/salt-bridge contacts plus an additional H-bond; its deprotonated carboxylate matches the highly cationic micro-environment, whereas the aromatic scaffold occupies a hydrophobic cavity within the binding site. MR retains the same aromatic surface but, because the carboxylate is esterified, it loses part of the Coulombic contribution; overall anchoring is therefore weaker, even though the desolvation penalties are somewhat mitigated. LG provides a broader array of H-bonds via its glucuronide, yet its bulkier, less planar shape limits penetration into the pocket and likely incurs a higher entropic cost upon binding. CAc, lacking both the “rosmarinic tail” and a second aromatic ring, engages essentially one salt bridge and one H-bond; with a smaller polar contact area and insufficient hydrophobic compensation, it yields the lowest predicted stability.

On Keap1-Nrf2, RAc shows hydrogen bonds to Leu365(B), Arg415(B), Ser602(B), and Val604(B), plus a salt bridge to Arg415(B) and a second salt bridge to Ser508(B). MR engages Ser383(A) and Tyr334(B) via hydrogen bonds, forming a π-cation contact with Arg415(B) and additional hydrogen bonds to Ser508(B) and Ser555(B). CAc displays two hydrogen bonds to Ser383(A) and Pro384(A). LG is predicted to π-stack with Tyr344(B), form a salt bridge to Arg415(B), and establish hydrogen bonds with Ser383(A), Asn387(A), Gln530(B), and Ser555(B) (Figure S2b). Within this cavity, H-bond density and polar complementarity appear decisive. LG forms a multivalent hydrogen-bond network, while the flavone core participates in π-stacking, and the glucuronide carboxylate establishes a salt bridge. This arrangement strikes an optimal balance between hydrophilic contacts and aromatic stacking. RAc also exploits a salt bridge, but the lower number of available donors/acceptors yields a sparser hydrogen-bond network than LG. MR, lacking a free carboxylate, forfeits part of the Coulombic anchoring that benefits RAc, though it still maintains a π-stacking interaction. CAc, which is much smaller, interacts almost exclusively through two hydrogen bonds; the reduced polar and hydrophobic surface explains its weak predicted affinity.

Finally, on NOX, RAc is predicted to hydrogen-bond to Ile160, Gly161, Val214, and Cys242, while interacting with Lys187 via hydrogen bonding and with Lys213 via a salt bridge. MR shows a single hydrogen bond to Asp179. CAc hydrogen-bonds to Ile160, Gly161, Gly244, and Pro298 and forms a salt bridge with Lys213. LG establishes hydrogen bonds with Gly158, Cys242, Ile243, and Pro298 and forms both hydrogen-bond and salt-bridge contacts with Lys187 and Lys213 (Figure S2c). The modelled site features two lysines (Lys187, Lys213) that act as an electrostatic “magnet” for acidic groups. LG engages both residues via a dual ionic anchor and supplements this with several H-bonds; the glucuronide greatly enlarges the polar surface, enhancing complementarity while its flexible glycosidic linkage still allows entry into the pocket. RAc preserves both salt bridges but offers a leaner H-bond set and an aromatic portion less suited to the lateral hydrophobic micro-cavities, resulting in somewhat lower, albeit still substantial, stabilisation. MR, being neutral, cannot capitalise on electrostatic anchoring; binding is driven almost exclusively by hydrogen bonding, and the pose remains closer to the pocket mouth. CAc, with a single salt bridge and few additional H-bond possibilities, pays in both electrostatics and contact area, placing it at the bottom of the ranking.

Although the specific lists of hydrogen bonds and salt bridges differ, every ligand in our series occupies the same functional pocket characterised by earlier studies. For iNOS, all four ligands cluster around the heme gate. In particular, RAc, MR, and CAc bind in the region described by Toraman et al. [83] and Merecz-Sadowska et al. [85]. Regarding Keap1-Kelch, our highest-scoring poses dock at the entrance of the pocket, precisely the site emphasised in multiple docking studies. Smaller ligands, such as CAc, move a few ångströms up toward the edge but remain within the previously mapped ETGE recognition groove. As far as NOX is concerned, the local backbone provides a comparable hydrogen bonding picture.

Because each docking protocol uses different parameters, residue-by-residue comparisons inevitably reveal minor discrepancies. What truly matters is the conserved pharmacophore. In the absence of high-resolution biophysical data (e.g., ITC, SPR, co-crystal structures, or extensive MD simulations), neither the published poses nor ours should be deemed “superior”; they represent complementary models that converge on the same binding region and collectively strengthen the mechanistic hypothesis.

Taken together, these qualitative patterns (π-stacking, salt-bridge/π-cation pairing, and multi-point hydrogen bonding) offer a structural rationale for the affinity ranking summarised in Table 3.

## 4. Conclusions

The aqueous fraction residual from *S. desoleana* steam distillation proved to be an interesting by-product with potential use. Moreover, it can be easily processed to be used in food supplements or healthy products due to its rich content of phenolic compounds. In particular, the most representative class of compounds is that of hydroxycinnamic acids, with RAc as the most abundant, followed by CAc and MR. Flavonoids are another important class of compounds, with LG as the most abundant one. The four extracts obtained from *S. desoleana* steam-distillation water residue showed antioxidant and antiradical activity, as confirmed by the assays thus performed (FRAP, CUPRAC, DPPH^•^, and ABTS^•+^). The total phenolic content was evaluated with Folin–Ciocalteu’s assay, confirming that the sample SEtOHA is the one with the highest phenol content, and it is also the one with the highest antioxidant and antiradical capacity. Furthermore, the two most active samples (SEtOHA and SH_2_OA) were evaluated for their cell viability, showing no toxic effects. On these two samples, a study was performed to investigate their protection against ROS-induced oxidation, showing significant results that were reflected in the counteraction of CyS oxidation. Furthermore, the SEtOHA extract has shown anti-inflammatory activity by inhibiting NO release in LPS-stimulated intestinal cells. Regarding the most representative phenolic compounds (RAc, MR, CAc, and LG), the in silico evaluation against three enzymes involved in the antioxidant response (iNOS, Keap1-Nrf2, and NOX) showed interesting results for both the numerical affinity ranking (Glide XP Docking Scores) and the predicted ligand binding models. However, the results remain sufficiently speculative to warrant further experimental studies.

In conclusion, taking all the results into account and considering further useful studies for a better understanding of the matrix used, *S. desoleana* steam-distillation water residue is a by-product with numerous possible future applications, especially in the nutraceutical, food, and pharmaceutical industries. Moreover, the exploitation of endemic plants can preserve rural landscapes, and promoting typical food consumption can contribute to a comprehensive healthy lifestyle typical of the Blue Zones.

## Figures and Tables

**Table 1:** Quantification of phenolic compounds by the LC-PDA method (mg/g dw).

Compounds	n°	SH_2_OA	SH_2_OB	SEtOHA	SEtOHB	SFH_2_O	SFEtOH
	MS ^§^	Mean ± sd	Mean ± sd	Mean ± sd	Mean ± sd	Mean ± sd	Mean ± sd
Total flavonoids		34.39 ± 1.27 ^a^	1.7 ± 0.26 ^b^	29.83 ± 1.29 ^c^	7.34 ± 0.13 ^d^	15.40 ± 1.08 ^e^	20.54 ± 1.84 ^f^
Luteolin 7-*O*-glucoside	**12**	3.08 ± 0.16 ^a^	0.22 ± 0.00 ^b^	3.26 ± 0.03 ^a^	1.24 ± 0.00 ^c^	nd	6.85 ± 0.69 ^d^
Luteolin glucuronide ^A^	**13**	11.04 ± 0.10 ^a^	0.27 ± 0.00 ^b^	7.33 ± 0.04 ^c^	nd	8.15 ± 0.57 ^d^	2.15 ± 0.23 ^e^
Isorhamnetin hexoside ^B^	**14**	1.15 ± 0.03 ^a^	0.04 ± 0.00 ^b^	0.77 ± 0.01 ^c^	nd	nd	0.10 ± 0.01 ^d^
Apigenin hexoside ^C^	**15**	0.33 ± 0.00 ^a^	nd	nd	nd	nd	0.15 ± 0.02 ^b^
Apigenin glucuronide ^C^	**17**	5.05 ± 0.05 ^a^	0.20 ± 0.00 ^b^	4.05 ± 0.01 ^c^	0.49 ± 0.00 ^d^	3.01 ± 0.29 ^e^	4.52 ± 0.50 ^c^
Hispidulin glucuronide ^D^	**18**	5.30 ± 0.17 ^a^	0.18 ± 0.00 ^b^	4.05 ± 0.02 ^c^	0.25 ± 0.00 ^d^	2.99 ± 0.24 ^e^	3.92 ± 0.35 ^f^
Luteolin	**21**	1.07 ± 0.06 ^a^	0.11 ± 0.00 ^b^	1.34 ± 0.09 ^c^	0.75 ± 0.00 ^d^	nd	nd
Isorhamnetin	**22**	0.94 ± 0.02 ^a^	0.08 ± 0.00 ^b^	0.86 ± 0.40 ^a^	0.47 ± 0.00 ^a^	nd	nd
Apigenin acetyl-glucoside ^C^	**23**	nd	nd	nd	0.06 ± 0.09 ^a^	nd	nd
Apigenin	**24**	0.54 ± 0.01 ^a^	0.07 ± 0.00 ^b^	0.69 ± 0.02 ^c^	0.40 ± 0.00 ^d^	nd	nd
Dimethyl-quercetin ethere ^A^	**25**	1.23 ± 0.01 ^a^	0.14 ± 0.00 ^b^	1.67 ± 0.01 ^c^	0.94 ± 0.01 ^d^	nd	nd
Hispidulin	**26**	1.15 ± 0.01 ^a^	0.16 ± 0.00 ^b^	1.74 ± 0.08 ^c^	1.07 ± 0.02 ^d^	nd	nd
Cirsimaritin ^D^	**27**	0.52 ± 0.17 ^a^	0.08 ± 0.00 ^b^	0.69 ± 0.30 ^a^	0.41 ± 0.01 ^a^	nd	0.45 ± 0.04 ^a^
Genkwain ^D^	**28**	0.46 ± 0.00 ^a^	0.06 ± 0.00 ^b^	0.56 ± 0.00 ^c^	0.32 ± 0.00 ^d^	nd	0.39 ± 0.04 ^e^
Other Flavonoids		2.53 ± 0.48 ^a^	0.14 ± 0.00 ^b^	2.81 ± 0.28 ^a^	0.95 ± 0.00 ^c^	1.25 ± 0.13 ^d^	2.01 ± 0.12 ^a^
Total hydroxycinnamic acids		361.27 ± 15.16 ^a^	25.10 ± 0.23 ^b^	518.64 ± 5.82 ^c^	197.09 ± 0.43 ^d^	158.69 ± 7.93 ^e^	57.35 ± 3.44 ^f^
Caffeic acid	**7**	15.33 ± 0.19 ^a^	1.19 ± 0.01 ^b^	26.69 ± 0.49 ^c^	11.86 ± 0.00 ^d^	nd	17.04 ± 1.02 ^e^
Ferulic acid	**11**	7.20 ± 5.04 ^a^	0.65 ± 0.01 ^b^	15.31 ± 0.75 ^c^	2.37 ± 0.01 ^d^	1.32 ± 0.07 ^e^	3.54 ± 0.21 ^a^
Rosmarinic acid	**16**	284.01 ± 4.43 ^a^	22.10 ± 0.20 ^b^	427.42 ± 1.99 ^c^	159.22 ± 0.26 ^d^	128.01 ± 11.52 ^e^	nd
Methyl rosmarinate ^E^	**20**	10.78 ± 0.05 ^a^	1.01 ± 0.01 ^b^	17.42 ± 1.03 ^c^	7.66 ± 0.03 ^d^	nd	10.25 ± 0.72 ^a^
Others hydroxycinnamic acids		43.95 ± 5.45 ^a^	0.15 ± 0.00 ^b^	31.79 ± 1.57 ^c^	16.00 ± 0.12 ^d^	25.36 ± 1.78 ^e^	20.52 ± 1.23 ^f^
Total hydroxybenzoic acids		3.93 ± 0.92 ^a^	0.22 ± 0.02 ^b^	5.30 ± 0.35 ^c^	1.49 ± 0.03 ^d^	2.71 ± 0.16 ^e^	3.28 ± 0.26 ^a^
Salvianic acid A (danshensu) ^F^	**4**	0.11 ± 0.00 ^a^	nd	0.16 ± 0.01 ^b^	0.15 ± 0.00 ^b^	0.10 ± 0.00 ^c^	nd
Protocatechuic acid hexoside ^F^	**3**	0.17 ± 0.01 ^a^	nd	0.24 ± 0.00 ^b^	0.12 ± 0.00 ^c^	0.09 ± 0.01 ^d^	0.12 ± 0.01 ^c^
Syringic acid	**10**	0.44 ± 0.21 ^a^	nd	0.44 ± 0.03 ^a^	0.62 ± 0.00 ^b^	nd	nd
Others		3.21 ± 0.69 ^a^	0.22 ± 0.02 ^b^	4.46 ± 0.31 ^c^	0.60 ± 0.02 ^d^	1.52 ± 0.14 ^e^	2.16 ± 0.15 ^f^
Total other compounds		0.04 ± 0.00 ^a^	nd	0.05 ± 0.00 ^b^	0.01 ± 0.00 ^c^	0.02 ± 0.00 ^d^	0.04 ± 0.00 ^a^
Homovanillic acid	**6**	0.04 ± 0.00 ^a^	nd	0.05 ± 0.00 ^b^	0.01 ± 0.00 ^c^	0.02 ± 0.00 ^d^	0.04 ± 0.00 ^a^
Total phenols		399.64 ± 17.36 ^a^	27.07 ± 0.28 ^b^	553.82 ± 7.47 ^c^	205.93 ± 0.59 ^d^	176.82 ± 12.38 ^e^	81.21 ± 4.87 ^f^

^§^ Peak number as reported in Table S1. ^A^ Expressed as luteolin equivalents; ^B^ expressed as isorhamnetin equivalents; ^C^ expressed as apigenin equivalents; ^D^ expressed as hispidulin equivalents; ^E^ expressed as rosmarinic acid equivalents; ^F^ expressed as syringic acid equivalents. nd: not detected. Data are given as mean ± SD (*n* = 3). Mean values within a line with different letters are significantly different (homogenous groups) at *p* ≤ 0.05.

**Table 2:** Total phenolic content and antioxidant activity of *S. desoleana* steam-distillation water residue.

Sample Code	TP ^A^	CUPRAC ^B^	FRAP ^B^	DPPH^• C^	ABTS^•+ C^
	(mg GAE/g dw)	(mmol Fe^2+^/g dw)		(mmol TEAC/g dw)	
SH_2_OA	75.28 ± 1.97 ^a^	4.10 ± 0.04 ^a^	2.04 ± 0.05 ^a^	0.63 ± 0.02 ^a^	0.75 ± 0.02 ^a^
SH_2_OB	3.52 ± 0.22 ^b^	0.15 ± 0.00 ^b^	0.08 ± 0.00 ^a^	0.02 ± 0.00 ^b^	0.03 ± 0.00 ^b^
SEtOHA	106.02 ± 6.02 ^c^	7.57 ± 0.05 ^c^	4.04 ± 0.14 ^c^	0.97 ± 0.03 ^c^	1.29 ± 0.02 ^c^
SEtOHB	32.44 ± 1.93 ^d^	1.42 ± 0.12 ^d^	0.84 ± 0.06 ^d^	0.13 ± 0.01 ^d^	0.23 ± 0.02 ^d^

^A^ The total phenolic content (TP) value is expressed as milligrams of gallic acid equivalent (GAE). ^B^ FRAP and CUPRAC values are expressed as the millimolar concentration of Fe^2+^, obtained from a dilution of FeSO_4_ having an equivalent antioxidant capacity to that of the extract. ^C^ DPPH^•^ and ABTS^•+^ values are expressed as the millimolar concentration of TEAC, obtained from a Trolox solution having an antiradical capacity equivalent to that of the extract; nm: not measurable. All values are expressed as mean ± SD (*n* = 3), and mean values within a column with different letters are significantly different at *p* ≤ 0.05.

**Table 3:** GlideXP docking scores (kcal/mol).

Compound	iNOS	Keap1-Nrf2	NOX
Rosmarinic acid	−7.901	−9.655	−9.026
Methyl rosmarinate	−6.809	−8.191	−7.478
Caffeic acid	−4.224	−5.122	−5.412
Luteolin-7-*O*-glucuronide	−4.864	−11.649	−11.696

## Data Availability

The original contributions presented in the study are included in the article and Supplementary Materials; further inquiries can be directed to the corresponding authors.

## Supplementary Materials

The following supporting information can be downloaded at: https://www.mdpi.com/article/10.3390/foods14132365/s1. Figure S1: (HR) LC-ESI-QTOF MS total compound chromatogram of *S. desoleana* water residue extract (sample SEtOHA) acquired in negative ion mode. Chromatographic conditions are described in the text. Peak identification is given in Table S1; Figure S2a: Three-dimensional and 2D representations of (A) rosmarinic acid, (B) methyl rosmarinate, (C) caffeic acid, and (D) luteolin-7-*O*-glucuronide in the iNOS active site (4NOS). Hydrogen bonds are depicted as dashed yellow lines, salt bridges as dashed magenta lines, π-cation interactions as dashed green lines, and π-π interactions as dashed blue lines. For panel D (2D representation), refer to the legend provided below the 2D image; Figure S2b: 3D and 2D representations of (A) rosmarinic acid, (B) methyl rosmarinate, (C) caffeic acid, and (D) luteolin-7-*O*-glucuronide in the Keap1-Nrf2 active site (4L7B). Hydrogen bonds are depicted as dashed yellow lines, salt bridges as dashed magenta lines, π-cation interactions as dashed green lines, and π-π interactions as dashed blue lines. For panel D (2D representation), refer to the legend provided below the 2D image; Figure S2c: 3D and 2D representations of (A) rosmarinic acid, (B) methyl rosmarinate, (C) caffeic acid, and (D) luteolin-7-*O*-glucuronide in the NOX active site (2CDU). Hydrogen bonds are depicted as dashed yellow lines, salt bridges as dashed magenta lines, π-cation interactions as dashed green lines, and π-π interactions as dashed blue lines. For panel D (2D representation), refer to the legend provided below the 2D image; Table S1: Compound identification by (HR) LC-ESI-QTOF MS/MS in *S. desoleana* water residue extract.

## Abbreviations

The following abbreviations are used in this manuscript:

AA	Antioxidant activity
ABTS^•+^	2,2-azinobis-(3-ethylbenzothiazoline-6-sulphonic acid
CAc	Caffeic acid
CUPRAC	Cupric-ion-reducing antioxidant capacity
CyS	Cysteine
CySS	Cystine
dp	Dry plant residue
DPPH^•^	2,2-diphenyl-1-picrylhydrazyl radical
dw	Dry weight
FRAP	Ferric reducing antioxidant power
GAE	Gallic acid equivalent
GSH	Reduced glutathione
GSSG	Oxidised glutathione
LG	Luteolin-7-*O*-glucuronide
MR	Methyl rosmarinate
NO	Nitric oxide
RAc	Rosmarinic acid
ROS	Reactive oxygen species
TBH	Tert-butyl hydroperoxide
TP	Total polyphenol
TPTZ	2,4,6-tris(pyridin-2-yl)-1,3,5-triazine
