# Supplementary material for: Salvia desoleana Atzei et Picci Steam-Distillation Water By-Products as a Source of Bioactive Compounds with Antioxidant Activities
Source: Foods. 2025 Jul 3;14(13):2365. doi: 10.3390/foods14132365 (PMC12249583; doi:10.3390/foods14132365)
Supplement: Supplementary file 1 [file foods-14-02365-s001.zip › foods-3693451-supplementary.pdf]

Salvia desoleana Atzei et Picci Steam-Distillation Water By-Products as a Source of Bioactive Compounds with Antioxidant Activities

Valentina Masala [1], Gabriele Serreli [2], Antonio Laus [3], Monica Deiana [2], Adam Kowalczyk [4] and Carlo Ignazio Giovanni Tuberoso [1]*

[1] Department of Life and Environmental Sciences, University of Cagliari, Cittadella Universitaria di Monserrato, S.P. Monserrato-Sestu km 0.700, 09042 Monserrato, Italy
[2] Department of Biomedical Sciences, University of Cagliari, Cittadella Universitaria di Monserrato, S.P. Monserrato-Sestu km 0.700, 09042 Monserrato, Italy
[3] Department of Life Sciences - University of Modena and Reggio Emilia, Italy
[4] Department of Pharmacognosy and Herbal Medicines, Faculty of Pharmacy, Wroclaw Medical University, Poland

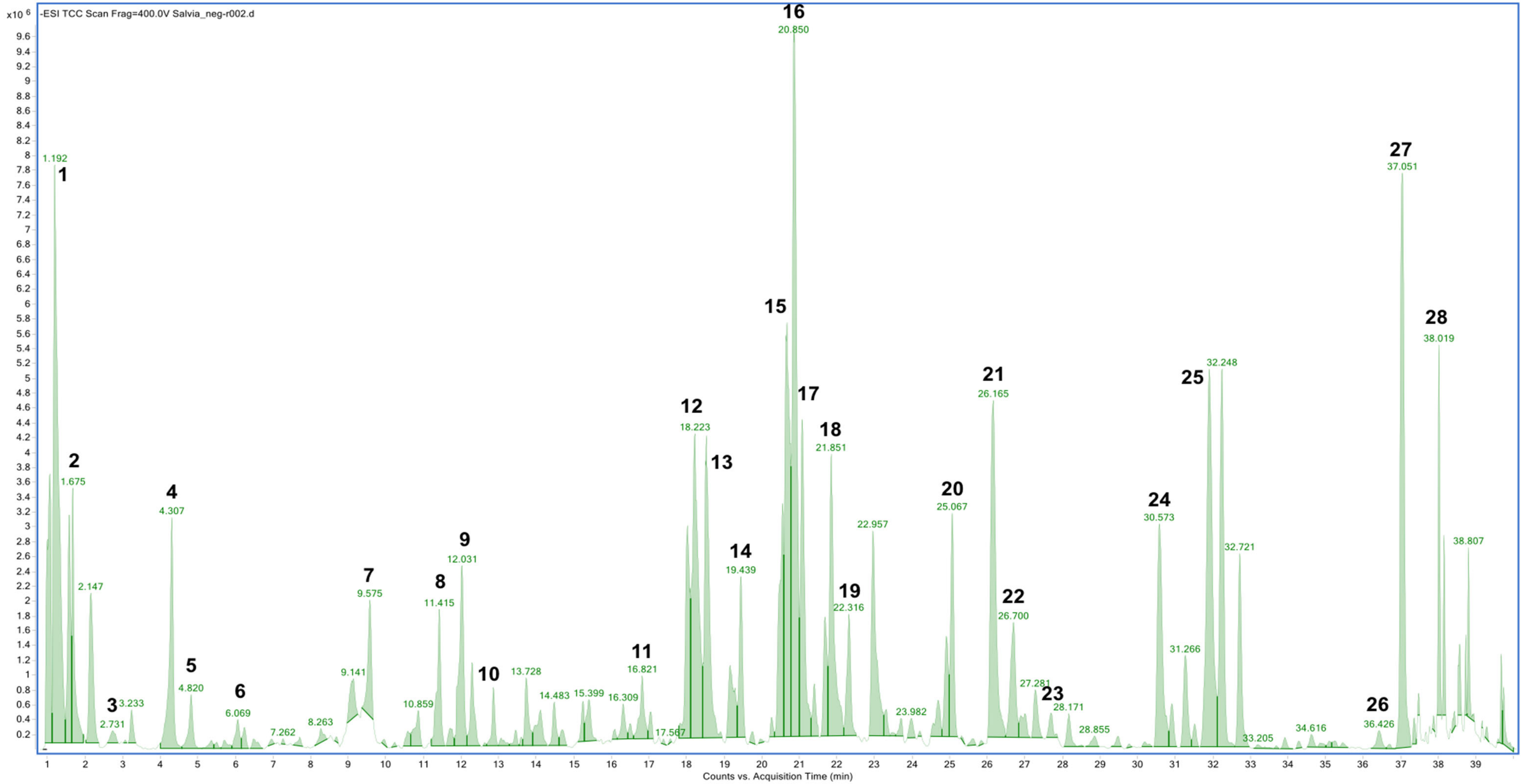

Figure S1. (HR) LC-ESI-QTOF MS total compound chromatogram of *S. desoleana* water residue extract (sample SEtOHA) acquired in negative ion mode. Chromatographic conditions are described in the text. Peak identification is given in Table S1.

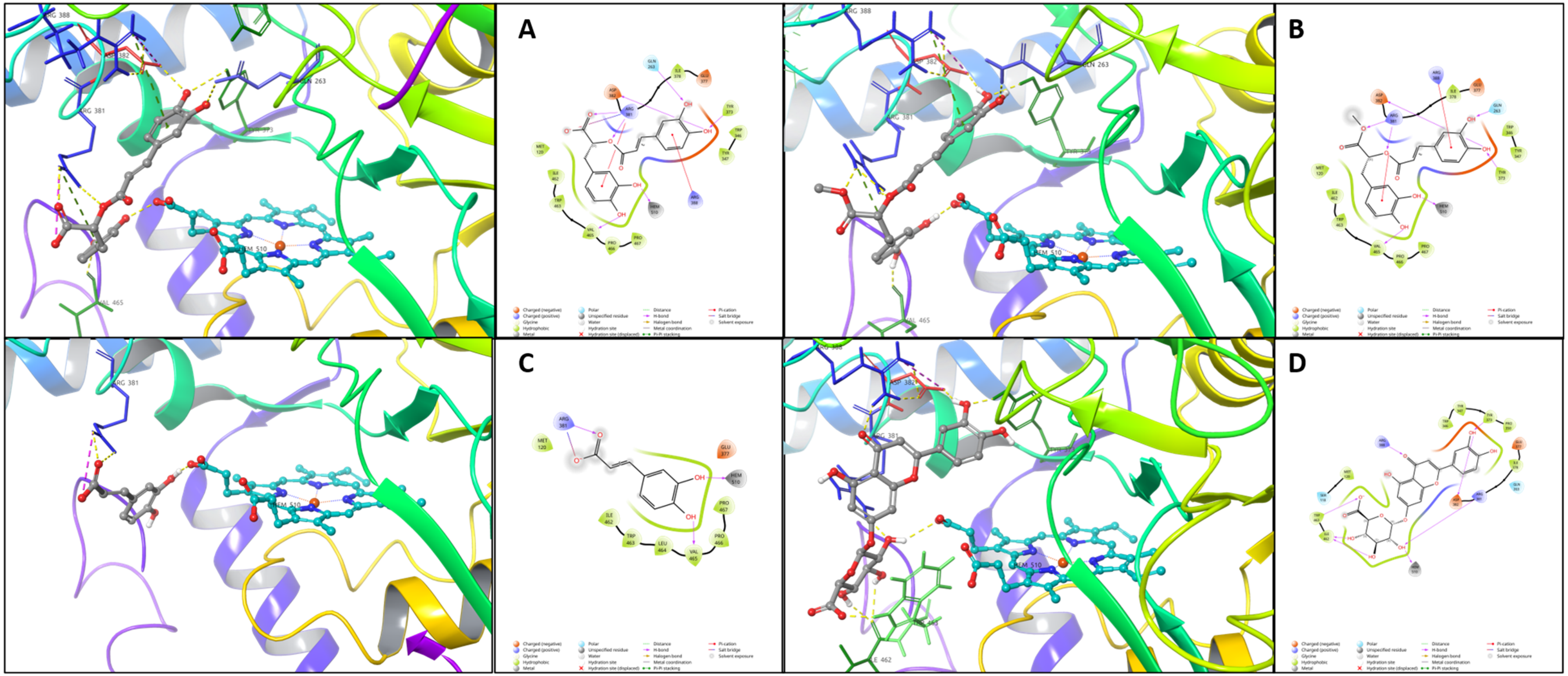

Figure S2a. Three-dimensional and 2D representations of (A) rosmarinic acid, (B) methyl rosmarinate, (C) caffeic acid, and (D) luteolin-7-*O*-glucuronide in the iNOS active site (4NOS). Hydrogen bonds are depicted as dashed yellow lines, salt bridges as dashed magenta lines, π-cation interactions as dashed green lines, and π-π interactions as dashed blue lines. For panel D (2D representation), refer to the legend provided below the 2D image.

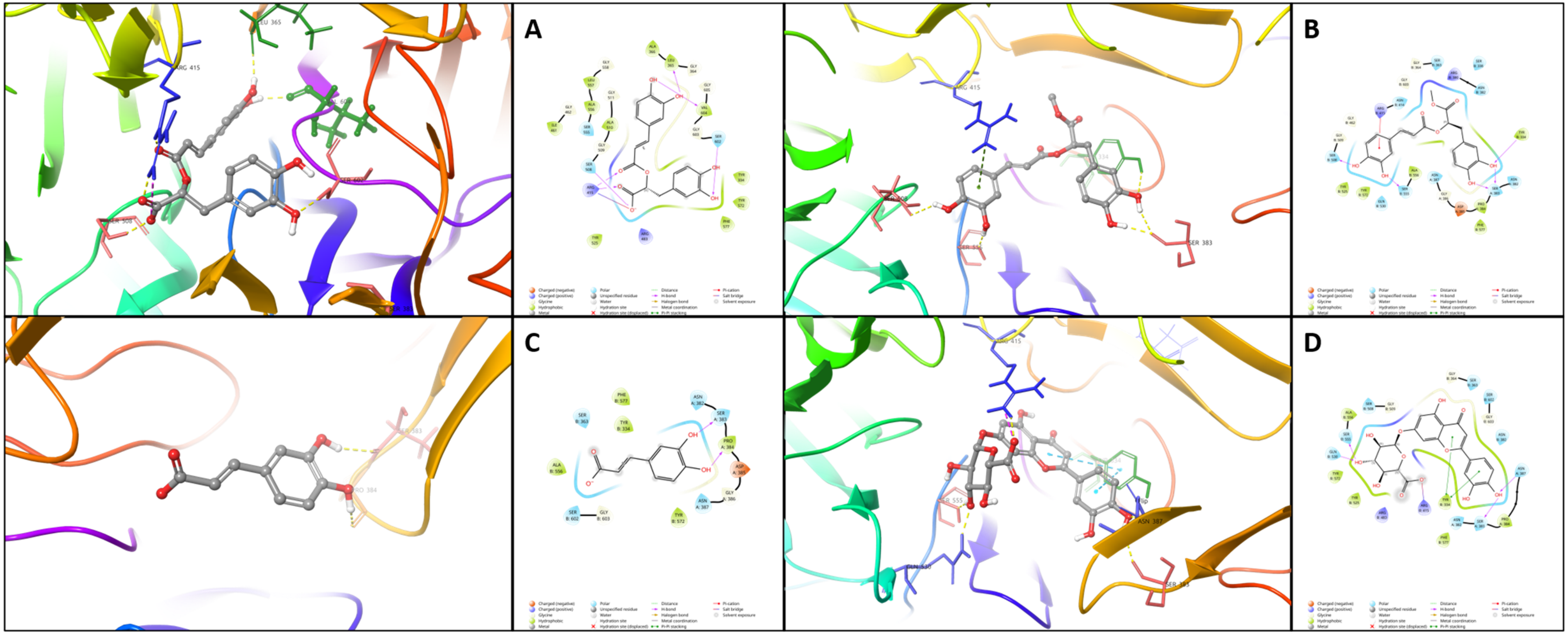

Figure S2b. Three-dimensional and 2D representations of (A) rosmarinic acid, (B) methyl rosmarinate, (C) caffeic acid, and (D) luteolin-7-*O*-glucuronide in the Keap1-Nrf2 active site (4L7B). Hydrogen bonds are depicted as dashed yellow lines, salt bridges as dashed magenta lines, π-cation interactions as dashed green lines, and π-π interactions as dashed blue lines. For panel D (2D representation), refer to the legend provided below the 2D image.

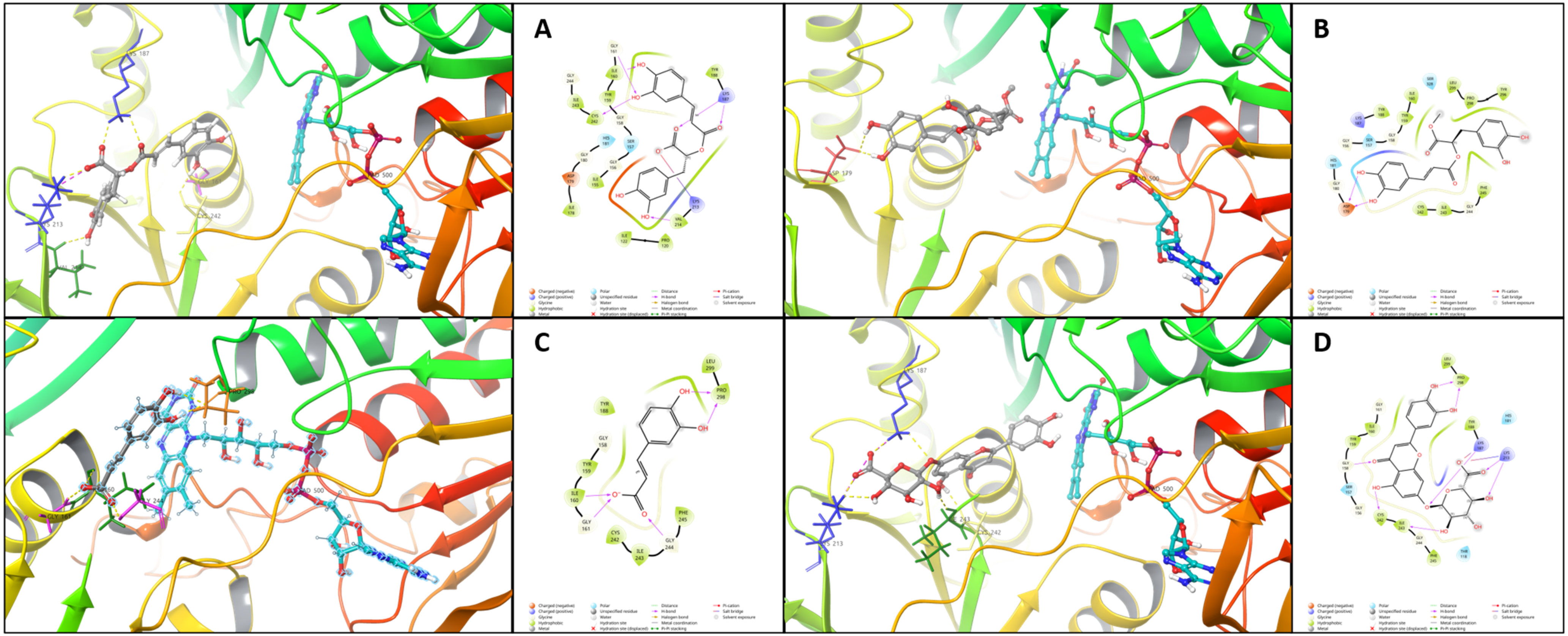

Figure S2c. Three-dimensional and 2D representations of (A) rosmarinic acid, (B) methyl-rosmarinate, (C) caffeic acid, and (D) luteolin-7-*O*-glucuronide in the NOX active site (2CDU). Hydrogen bonds are depicted as dashed yellow lines, salt bridges as dashed magenta lines, pi-cation interactions as dashed green lines, and π-π interactions as dashed blue lines. For panel D (2D representation), refer to the legend provided below the 2D image.

Table S1. Compound identification by (HR) LC-ESI-QTOF MS/MS in *S. desoleana* water residue extract.

n°	Rt min	Identity	$[M-H]^-$ *m/z*	molecular formula	Δ ppm	MS/MS[a] *m/z*	$[M+H]^+$ *m/z*	Δ ppm	MS/MS[a] *m/z*	References [b]	Level [c]	*S. desoleana* extract [d]			
												SFH_2O	SFEtOH	SH_2OA	SEtOHA
1	1.29	Citric acid	191.0201	$C_6H_8O_7$	-1.47	87.0091(100)/111.0085(52)	193.0456	0.46	-	[44,45]	1	x	x	x	x
2	1.69	Fumaric acid	115.0701	$C_4H_4O_4$	-0.05	-	117.0583	1.35	-	[46,47]	1	x	x	x	x
3	2.73	Protocatechuic acid hexoside	315.0731	$C_{13}H_{16}O_9$	0.72	152.0106(100)/108.0211(66)	-	-	-	[48]	2	x	x	x	x
4	4.31	Danshensu	197.1700	$C_9H_{10}O_5$	-0.43	135.0446(100)/123.0447(81)	221.0419[Na^+]	-0.83	-	[49]	2	x	-	x	x
5	4.82	Tryptophan	203.0827	$C_{11}H_{12}N_2O_2$	-0.25	116.0506(100)	227.0793 [Na^+]	1.23	146.0602(100)/118.0646(71)	[50]	1	x	x	x	x
6	6.07	Homovanillic acid	181.0879	$C_9H_{10}O_4$	0.51	135.0445(100)	183.0976	1.45	-	[51]	1	x	x	x	x
7	9.57	Caffeic acid	179.0367	$C_9H_8O_4$	-0.92	179.03(6)/135.04(100)	181.0497	0.66	153.0540(100)/65.0292(51)	[52-55]	1	-	x	x	x
8	11.42	Tuberonic acid glucoside	387.1709	$C_{18}H_{28}O_9$	3.19	207.10(35)/59.01(100)	411.1628 [Na^+]	0.55	85.0634(100)/209.1199(96)/191.1069(83)	[55]	2	x	x	x	x
9	12.031	Salvianic acid C	377.9070	$C_{18}H_{17}O_9$	4.11	161.0231(15)/179.0227(10)	-	-	-	[45,56]	2	x	x	x	x
10	12.89	Syringic acid	197.0459	$C_9H_{10}O_5$	0.35	182.0205(100)/138.0321(31)	199.0598	-0.83	-	[57,58]	1	-	-	x	x
11	16.82	Ferulic acid	193.0511	$C_{10}H_{10}O_4$	0.27	134.0358(100)/89.0397(14)	195.0649	-1.27	-	[45]	1	x	x	x	x
12	18.22	Luteolin-7-*O*-glucoside	447.0946	$C_{21}H_{20}O_{11}$	-0.67	285.0410(100)/284.0330(43)	449.1089	3.59	287.0560(100)	[52,55, 59]	1	-	x	x	x
13	18.678	Luteolin -7-O-glucuronide	461.0967	$C_{21}H_{18}O_{12}$	1.79	285.0410(100)/	463.0876	0.93	287.0553(100)	[59]	2	x	x	x	x
14	19.44	Isorhamnetin hexoside	477.1062	$C_{22}H_{21}O_{12}$	2.14	315.0502(100)/299.0208(94)	501.1002[Na^+]	0.77	317.0663(100)/302.0416(17)	[45,56]	2	-	x	x	x
15	20.52	Apigenin hexoside	431.1002	$C_{21}H_{19}O_{10}$	2.67	269.0440(41)/268.0374(100)	433.1138	1.97	271.0604(100)	[45,59]	2	-	x	x	-

16	20.85	Rosmarinic acid	359.0780	$C_{18}H_{16}O_8$	-3.09	161.0241(100)/197.0449(15)/179.0344(17)	383.0737[Na^+]	-0.06	163.0387(100)/135.0446(38)	[52,54,55,57-59]	1	x	-	x	x
17	21.07	Apigenin glucuronide	445.8967	$C_{21}H_{18}O_{11}$	0.39	269.0457(100)	447.0936	2.17	271.0610(100)	[59]	2	x	x	x	x
18	21.85	Hispidulin glucuronide	475.3450	$C_{22}H_{22}O_{11}$	0.59	299.0563(100)/284.0328(33)	477.1044	2.50	301.0710(100)	[59]	2	x	x	x	x
19	22.35	Salvianolic acid K	555.1161	$C_{27}H_{24}O_{13}$	0.97	161.0243(100)/135.0449(99)	579.1103[Na^+]	-0.16	-	[44,55]	2	x	x	x	x
20	25.067	Methyl rosmarinate	373.2098	$C_{19}H_{18}O_8$	2.32	135.0455(100)/175.0405(48)	397.0894 [Na^+]	0.86	145.0293(100)/117.0324(42)	[44,54]	2	-	x	x	x
21	26.17	Luteolin	285.0418	$C_{15}H_{10}O_6$	4.73	-	287.0560	1.02	-	[53,55,57,58]	1	-	-	x	x
22	26.70	Isorhamnetin	315.0978	$C_{16}H_{12}O_7$	4.55	300.0283(100)	339.0477 [Na^+]	0.59	302.0426(100)	[44,60]	1	-	-	x	x
23	27.83	Apigenin acetyl-glucoside	473.1103	$C_{23}H_{22}O_{11}$	1.11	269.0454(100)/268.0370(62)	475.1234	-0.19	271.0605(100)	[61]	2	-	-	-	x
24	30.57	Apigenin	269.0468	$C_{15}H_{10}O_5$	4.71	-	271.0609	2.76	-	[52,53,55,58]	1	-	-	x	x
25	31.91	Dimethyl quercetin ether	329.0683	$C_{17}H_{14}O_7$	1.65	299.0207(100)/314.0453(40)	331.0818	2.01	298.0472(47)/316.0573(29)	[48,56]	2	-	-	x	x
26	36.43	Hispidulin	299.0572	$C_{16}H_{12}O_6$	2.38	284.0319(100)	301.0709	0.90	286.0471(100)	[62]	1	-	-	x	x
27	37.051	Cirsimaritin	313.0987	$C_{17}H_{14}O_6$	2.56	283.0251(100)	315.0865	0.45	282.0536(41)/300.0620(22)	[59,44]	2	-	x	x	x
28	38.16	Genkwanin	283.0622	$C_{16}H_{12}O_5$	3.56	268.0387(100)	285.0765	0.73	-	[45]	2	-	x	x	x

[a] In parentheses, the relative intensity; [b] references numbered as reported in the text of the manuscript; [c] according to Blaženović [43]; [d] x = detected, - = not detected.
